# Facultative predation expands the ecological repertoire of *Streptomyces*

**DOI:** 10.1128/mbio.00563-26

**Published:** 2026-04-20

**Authors:** Keith Yamada, Arina Koroleva, Heli Tirkkonen, Vilja Siitonen, Mitchell Laughlin, Amanda Moglia, Soheila Matroodi, Amir Akhgari, Guillaume Mazurier, Jarmo Niemi, Mikko Metsä-Ketelä

**Affiliations:** 1 Department of Life Technologies, University of Turku 8058, Turku, Finland; University of Strathclyde, Glasgow, United Kingdom

**Keywords:** *Streptomyces*, yeast, catabolic enzymes, polyenes, predation

## Abstract

Microbial predators obtain energy from killing other living cells. *Streptomyces* are soil bacteria that are known to produce numerous catabolic enzymes and antimicrobial compounds to defend against competing organisms. Here, we demonstrate that *Streptomyces* are predatory bacteria that prey on *Saccharomyces cerevisiae*. Time-lapse fluorescence microscopy and scanning electron microscopy revealed that predation is initiated by physical contact between *Streptomyces lavendulae* YAKB-15 and yeast cells. Comparative transcriptomics indicated that the interaction triggered the production of numerous extracellular catabolic enzymes and natural products, while delaying morphological development. Proteomics and enzyme assays confirmed co-culture-dependent production of carbohydrate-active enzymes (CAZymes), including various glucanases, mannosidases, and chitinases, which degraded the yeast cell wall. *Streptomyces lavendulae* YAKB-15 destabilized yeast cell membranes through the production of two polyene antifungal agents, pentamycin and filipin III. We found that the bioactivity was enhanced by cell-associated cholesterol oxidase ChoD, putatively by modulating sterol extraction kinetics. Metabolomic analyses suggest *Streptomyces* assimilates yeast cell sterols as nutrients. Furthermore, we observed the depletion of yeast-derived phosphatidylcholine and phosphatidylethanolamine lipids, which points to their consumption. We show that yeast predation is a common phenomenon in *Streptomyces*, which changes the paradigm of how these bacteria should be considered in the soil microbiome ecosystem.

**IMPORTANCE:**

Soil is a rich environment for microbes, where they compete for space and resources. *Streptomyces* bacteria are well known for their ability to synthesize natural products, particularly antibiotics, that are used in chemical defense against competing microbes. Here, we show that *Streptomyces* are, in fact, predatory bacteria. Upon encountering yeast cells, *Streptomyces* initiate the production of numerous enzymes that digest the cell wall and cell membrane. In addition, the interaction triggers the production of natural products that destabilize the yeast cell membrane. Collectively, these actions lead to the death of yeast cells and release of cellular building blocks that *Streptomyces* can use as nutrients. The work fundamentally shifts the paradigm of how *Streptomyces* are perceived within the soil microbiome ecosystem.

## INTRODUCTION

The soil microbiome is one of the most biologically diverse communities in the biosphere (1), where numerous bacteria, fungi, archaea, and protists compete for space and resources (2). Soil is a relatively nutrient scarce environment where carbon is trapped in complex polysaccharides, where nitrogen concentrations vary and minerals, such as iron, are limited (1). In addition, fluctuations in temperature, pH, salinity, and water availability induce abiotic stresses that challenge the survival of microbes in soil (3). Despite these challenges, microorganisms are overwhelmingly abundant in soils and represent the largest fraction of global biomass on Earth after plants (4). Bacteria (15% of total biomass) and fungi (2%) are the predominant microorganisms in soil and contribute more to the biomass than archaea and protists. Estimates suggest that 1 g of soil may harbor up to 10^11^ bacteria and 200 m fungi (5).

*Streptomyces* are one of the most prevalent genera of Actinobacteria, which play a significant role in the soil microbiome (4). These Gram-positive, multicellular bacteria have a complex life cycle that enables adaptation to environmental stress. Under unfavorable conditions, *Streptomyces* form spores that survive long periods, while nutrient availability triggers germination and vegetative mycelium growth. *Streptomyces* are integrated into the global carbon cycle and produce various hydrolytic enzymes to catabolize plant biomass constituents, including cellulose, hemicellulose, and lignin (6, 7). Another abundant carbon source for *Streptomyces* is chitin, which originates from exoskeletons of insects and crustaceans, and cell walls of fungi. Degradation of these natural polysaccharides typically requires an array of extracellular Carbohydrate-Active enZYmes (CAZymes) with different substrate specificities to break down complex mixtures of biopolymers (8). In addition, *Streptomyces* excel in their ability to harness insoluble ferrous iron from the soil through the biosynthesis of iron chelating siderophores and uptake systems for the siderophores-iron complexes (9–11).

Upon nutrient depletion, *Streptomyces* develop into aerial hyphae and ultimately into spores. *Streptomyces* are best known for their ability to synthesize complex biologically active natural products that have found use in the clinic as antimicrobials, anticancer agents, and immunosuppressants (12). Indeed, *Streptomyces* genomes harbor a high number of diverse biosynthetic gene clusters (BGCs), which are responsible for the production of bioactive secondary metabolites. Classically, antibiotic production is associated with the onset of sporulation, with bioactive compounds being secreted to defend against competing organisms (13). However, more recently, it has become accepted that *Streptomyces* use a multitude of extracellular mechanisms to detect and respond to the presence of competing microbes (7). Close physical contact (14–17) and various signaling molecules (7) in the soil microbiome have been shown to initiate cascades of reactions that elicit the production of secondary metabolites (16, 18). The natural products of *Streptomyces* are often associated with amensalistic killing during morphological differentiation, where one organism harms another without cost or benefit.

In contrast, in antagonistic microbe-microbe interactions such as predation, the predatory bacteria not only kill their prey but also gain a benefit from the action through consumption of macromolecules as nutrients (19). Predatory bacteria are found in different phyla and have evolved diverse strategies to kill bacteria, including epibiotic and endobiotic mechanisms, where the prey cells are lysed either from the outside or inside, respectively. The predatory behaviors of the Gram-negative soil-dwelling myxobacteria have been well characterized, including gliding motility to find prey and secretion of antibiotics and bacteriolytic enzymes to assimilate target microbes (20, 21). *Streptomyces* have not classically been considered predatory bacteria even though evidence for predation has been observed in microbiological and environmental studies (22, 23). *Streptomyces* isolates have been shown to be able to grow on live cells of other bacteria including *Staphylococcus aureus*, *Bacillus* sp. (24), and cyanobacteria (25) when no other sources of nutrients are available. In addition, population studies in soil (23) and carbon uptake in natural microbial assemblages (22) have identified *Streptomycetaceae* as possible predatory bacteria.

Here, we demonstrate that *Streptomyces lavendulae* YAKB-15 (26) and related strains are able to prey on *Saccharomyces cerevisiae* in co-cultivations. We applied a combination of transcriptomics, metabolomics, and proteomics, natural product chemistry, enzymatic assays, and confocal fluorescence microscopy studies to investigate the molecular basis of predation. We demonstrate that physical contact elicits the production of antifungal agents and hydrolytic exoenzymes to target all major components of the yeast cell wall and the cell membrane. This work redefines the ecological role of *Streptomyces* as bacterial predators and provides a systems-level analysis of yeast assimilation as a nutrient source.

## RESULTS AND DISCUSSION

### Yeast cells are assimilated upon physical contact with *S. lavendulae* YAKB-15

*S. lavendulae* YAKB-15 is an industrial producer of cholesterol oxidase ChoD, where the production is dependent on the presence of *Sacc. cerevisiae* cells (26). We initiated our study with microscopic evaluation of *Streptomyces*-yeast co-cultures, which revealed that yeast cells adhered to *S. lavendulae* YAKB-15 mycelium in liquid flask cultures by day 1. Surprisingly, extended cultivations demonstrated disturbances in yeast cell morphology by day 7 and the ultimate disappearance of yeast cells in 20-day cultures (Fig. S1), indicating that biological macromolecules from yeast may have been utilized as nutrients by *S. lavendulae* YAKB-15.

**Fig 1:**
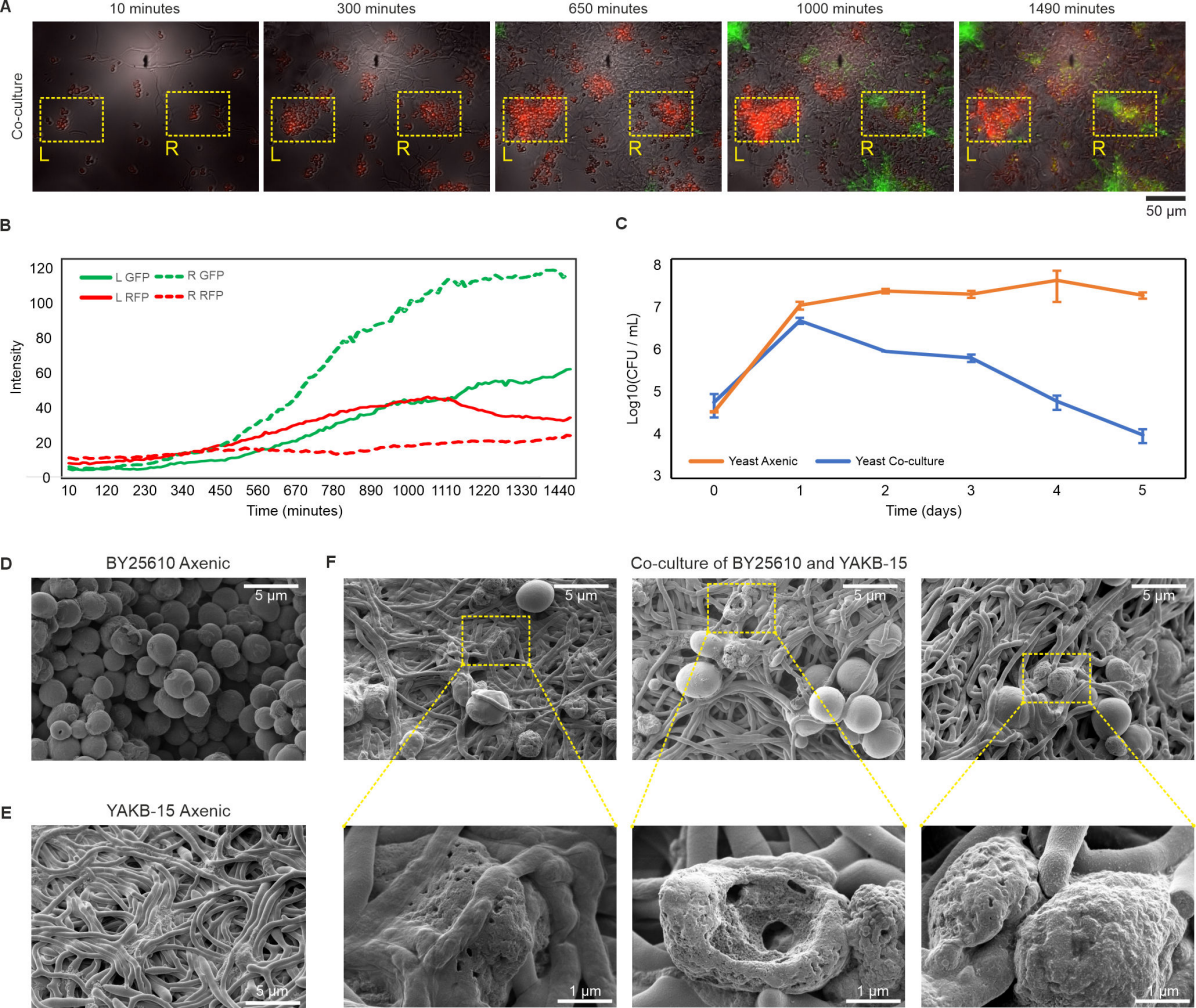
Microscopic and growth dynamics of *Streptomyces*–yeast interactions. (**A**) Time-lapse confocal fluorescence microscopy of *S. lavendulae* YAKB-15 (carrying a *choD*-GFP promoter probe) and *Sacc. cerevisiae* BY25610 (constitutively expressing mCherry/RFP) in co-culture over 1,490 min. Yellow boxes highlight two yeast populations: the left population (L) remained sequestered from *S. lavendulae*, while the right population (R) engaged in direct interaction. (**B**) Fluorescence intensity quantification of the populations shown in (**A**). (**C**) Yeast viability measured in Log_10_CFU/mL over 5 days, comparing growth in axenic culture (orange) vs co-culture with *S. lavendulae* (blue). Data points represent the mean ± SD (*n* = 3). (**D and E**) Scanning electron microscopy (SEM) of axenic *Sacc. cerevisiae* BY25610 (**D**) and *S. lavendulae* YAKB-15 (**E**). (**F**) SEM of the co-culture interaction. Insets provide high-magnification views of yeast cells exhibiting significant morphological degradation and cell-wall pitting upon contact with *S. lavendulae*.

In order to monitor individual cells, we turned to time-lapse confocal microscopy (Movie S1) to examine co-cultures of *S. lavendulae* YAKB-15/pS_GK_ChoD, which harbors a *choD* promoter probe plasmid for expression of green fluorescent protein (GFP), and *Sacc. cerevisiae* BY25610 (27), where red fluorescent protein (RFP) is constitutionally expressed. Still captures of a 1-day movie (Movie S1) recording revealed two yeast cell populations of approximately equivalent size at 10 min, which initially duplicated at an equal rate until 300 min (Fig. 1A, L and R insets). We observed *S. lavendulae* mycelium growing into one of the clusters of yeast cells (Fig. 1A, R inset), and a significant disturbance in yeast cell growth became visible at 650 min in comparison to yeast cells that are not in physical contact with *S. lavendulae* (Fig. 1A, L inset). Contact with *S. lavendulae* led to a drastic decrease in the RFP signal from yeast cells and the appearance of a GFP signal, indicating activation of ChoD production, only from *S. lavendulae* in physical contact with yeast at 1,000 min (Fig. 1A, L and R insets). The disappearance of yeast cells and the extension of the GFP signal to the entire mycelium network were evident at 1,490 min (Fig. 1A). The changes in yeast cell morphology and loss of red fluorescence were more clearly visible at higher digital magnifications (Movie S2). Analysis of fluorescence signal intensities of interacting and non-interacting cell populations from the same microscopy images demonstrated substantial changes with a 2.3-fold increase in GFP and a 2.3-fold decrease in RFP fluorescence in interacting populations at 1,100 min (Fig. 1B).

While microscopy provided visual evidence of contact-dependent lysis, we quantified the impact of yeast survival via colony-forming unit (CFUs) assays. Daily sampling indicated that both cultures contained an equal number of viable yeast cells on early time points on day 0 and day 1 (Fig. 1C). The influence of *Streptomyces* predation started to appear on day 2 and increased to a 2,212-fold difference on day 5 (Fig. 1C) in the number of viable cells in the axenic and co-culture samples. This noteworthy reduction in the number of viable yeast cells suggested that the interaction is not merely opportunistic scavenging but predation.

Next, we investigated the physical *Streptomyces*-yeast interactions in more detail at the cellular level by scanning electron microscopy (SEM) (Fig. 1D, E and F). After 3 days of *S. lavendulae* YAKB-15 and *Sacc. cerevisiae* BY25610 co-cultivation, we observed marked morphological alterations in yeast cells (Fig. 1F). Originally round or ovoid in shape, the yeast cells transformed into deformed, shrunken forms with irregular, roughened surfaces (Fig. 1F). Notably, we also observed holes and cavities in the yeast cells, suggesting structural degradation or lytic activity caused by *Streptomyces* (Fig. 1F). This type of damage was absent in yeast cells grown in monoculture (Fig. 1D).

### Comparative transcriptomics reveals an extensive predatome utilized by *S. lavendulae* YAKB-15 to prey on yeast

To gain more insight into the microbe-microbe interactions, we performed RNA-Seq from axenic and co-culture samples of *S. lavendulae* YAKB-15 and *Sacc. cerevisiae*. The transcriptomic experiments were performed under two conditions. First, *S. lavendulae* YAKB-15 was co-cultured with live yeast in synthetic complete (SC) medium. However, these experiments did not yield sufficient sequence reads from *S. lavendulae* YAKB-15 due to the abundance of yeast RNA in the samples (see below). Second, transcriptomics data were acquired by growing *S. lavendulae* YAKB-15 in Y medium containing 26 g/L autoclaved dead yeast cells. We surmised that autoclaved yeast would function as a suitable model system since we have previously shown that the intact cellular structures of autoclaved yeast cells are sufficient to induce expression of *choD*, which does not occur when yeast extract is used instead of autoclaved yeast cells (26). This experimental approach prevented growth of yeast cells and enabled collection of RNA-seq data from *Streptomyces*.

**Fig 2:**
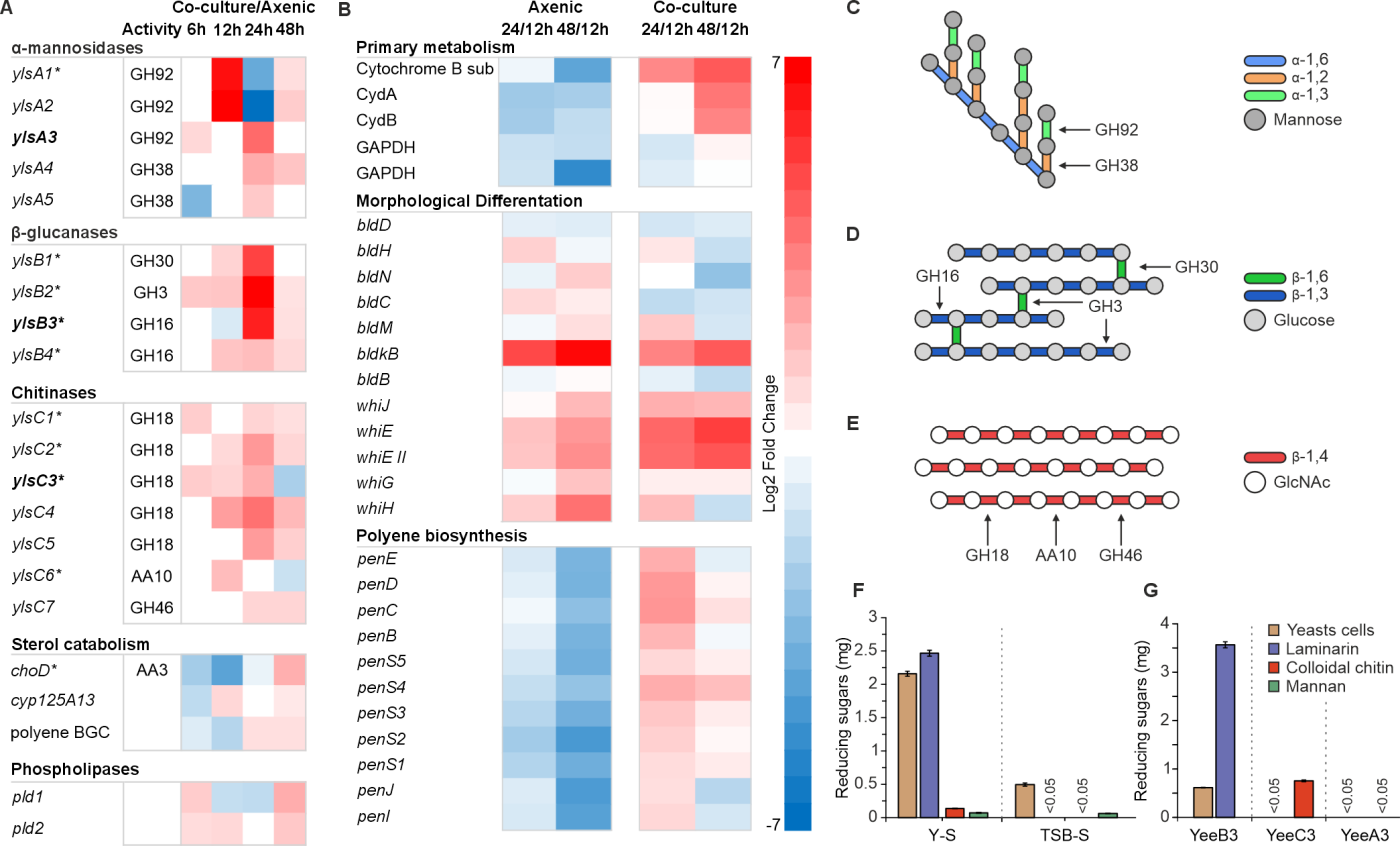
Bioinformatics, transcriptomics, and proteomics analysis of yeast predation. (**A**) Comparative transcriptomics profiling reveals differential expression of enzymes targeting yeast cell wall and cell membrane components, between axenic *S. lavendulae* cultures and *S. lavendulae* co-cultured with yeast cells. Gene products that were confirmed by proteomics at 24 h are marked with an asterisk (*) and heterologously expressed proteins are in bold, and their glycoside hydrolase (GH) families are shown. (**B**) Comparative transcriptomics profiling of classical developmental phase genes and the polyene genes between 12 h and 24 h or 48 h. Data analysis demonstrates that morphological differentiation is upregulated in axenic cultures, while in contrast primary metabolism and polyene biosynthesis are upregulated under co-culture conditions. (**C, D, and E**) Structures of yeast mannan, glucan, and chitin, respectively, and their glycosidic bonds. Hydrolysis of specific glycosidic bonds is shown with GH families. (**F**) Enzymatic activity of the *Streptomyces* secretome from cultures with whole autoclaved yeast cells (Y-S) and yeast-free (TSB-S) media, using whole autoclaved yeast cells and individual yeast cell wall components as substrates. (**G**) Enzymatic activities of YlsB3, YlsC3, and YlsA3 with whole autoclaved yeast cells and individual yeast cell wall components used as substrates. Error bars indicate the standard deviation of three technical replicates.

Comparative transcriptomics of *S. lavendulae* YAKB-15 from axenic cultures and in the presence of dead yeast cells in Y medium at four time points revealed an extensive predatome (28) in *Streptomyces* and uncovered changes in gene expression patterns of 185 of CAZyme genes (Fig. S2A), which encode enzymes capable of lysing and digesting the polysaccharide-rich yeast cell wall (29). We observed the upregulation of different glycoside hydrolase (GH) families of five α-mannosidases, four β-glucanases, and seven chitinases (Fig. 2A). Temporal control of CAZyme gene expression correlated well with enzymatic activities required for the digestion of yeast cell wall, with time-course analysis indicating an initial mean 16.1-fold upregulation of secreted exo-acting α-mannosidases of the GH92 family at 12 h, followed by subsequent upregulation of intracellular GH38 family α-mannosidases at 24 h (Fig. 2A and C).

In addition to gene products acting on the cell wall, we observed the upregulation of genes putatively influencing yeast cellular membranes. Genes related to catabolism of sterols, such as the flavoprotein *choD* and a putative P450 sterol monooxygenase (30), *cyp125A13*, were upregulated under co-culture conditions (Fig. 2A). Similarly, genes involved in phospholipid (31) catabolism, e.g., the putative phospholipases *pld1* and *pld2*, were upregulated (Fig. 2A).

The transcriptomics data also revealed large changes in general metabolism and morphological differentiation in *Streptomyces* upon microbe-microbe interactions. Under axenic conditions, time-course abundance difference analysis demonstrated the downregulation of indicator genes for primary metabolic processes such as oxidative phosphorylation (*cyd*) and glycolysis and glyconeogenesis (*gapdh*) (Fig. 2B). Furthermore, genes involved in morphological differentiation (*bld*) and sporulation (*whi*) (32) were upregulated (Fig. 2B), suggesting that *S. lavendulae* followed a canonical developmental cycle. This was in contrast to the co-culture samples, where the transcriptomic data indicated that *S. lavendulae* remained in a vegetative mycelium growth phase with upregulation of primary metabolism genes and delayed expression of genes involved in morphological differentiation (Fig. 2B).

In terms of secondary metabolism, the most coherent transcriptomics signal indicated the upregulation of an entire type I polyketide synthase BGC denoted as *pen* due to the similarity to known polyene gene clusters (33). The upregulation appeared to be related to yeast interactions since it occurred exclusively in co-culture samples at 24 h and the BGC remained dormant under axenic conditions (Fig. 2B). We also observed upregulation of the putative biosynthesis genes of the sesquiterpene geosmin (34) responsible for the characteristic odor of soil, which has been shown to act as an interspecies signaling molecule (35). In addition to the polyene and geosmin pathways, large changes in the biosynthesis of other unknown secondary metabolites (Fig. S2B) were observed that included both up- and downregulation. However, the unusual gene set compositions of these putative pathways precluded comprehensive bioinformatic analysis by antiSMASH (36) and prediction of the chemical structures of the metabolites. Five metabolic pathways appeared to be activated in response to yeast cells, most notably BGCs related to a copper chelating diisonitrile chalcophore SF2768-type siderophore (37), a foxicin-type ferric ion siderophore (38), and a stenothricin-type antibiotic (39) (Fig. S2B). In turn, six BGCs appeared to be silenced, including a herboxidiene-type anti-cancer polyketide (40) and a mirubactin-type siderophore (41).

Comparative transcriptomics performed with live yeast cells in SC medium facilitated our understanding of the response from *Sacc. cerevisiae*, which indicated that yeast could not effectively defend against the attack by *S. lavendulae*. The dominant response was gene downregulation (Fig. S3A), where the largest group of genes were related to cell wall biosynthesis, various stress responses (e.g., oxidative, formate, and pH), and strong 97-fold downregulation of ribosomal RNA (rRNA) and small nucleolar RNA (snoRNA) synthesis. Only four genes were upregulated in yeast, where the most interesting observation was upregulation of a polyamine transporter (Fig. S3B), which play a key role in the yeast stress response (42).

### Proteomics and enzyme activity assays confirm digestion of yeast cell wall components by *S. lavendulae* YAKB-15

To validate whether the extensive upregulation of CAZyme genes observed in our transcriptomic data translated into the physical secretion of active enzymes, we analyzed the extracellular proteome of the co-cultures via SDS-PAGE and mass spectrometry. We detected 9 out of the 16 upregulated CAZymes and the cholesterol oxidase ChoD when *S. lavendulae* YAKB-15 was grown in the presence of either autoclaved yeast cells in Y medium or live yeast cells in SC medium (Fig. 2A; Data S1).

We subsequently measured the degradation of yeast cell wall carbohydrate biopolymers by colorimetric analysis of reducing sugar content using DNS (3,5-dinitrosalicylic acid). To estimate the overall fragmentation of the yeast cell wall, we initially used whole autoclaved yeast cells as a substrate. Samples from the secretome of *S. lavendulae* YAKB-15 co-cultured with yeast displayed a 4.4-fold increase in total hydrolytic activity compared to samples from axenic *S. lavendulae* YAKB-15 cultures (Fig. 2F). To measure specific CAZyme activities, we utilized the storage glucan laminarin, colloidal chitin, and mannan as substrates. Co-culture samples harbored high glucanase and low chitinase and mannosidase activities in comparison to samples from axenic cultures (Fig. 2F).

The experiments were followed by activity assays with purified enzymes (Fig. 2G). We selected three enzymes from this putative yeast lytic system for heterologous protein production in *Escherichia coli*: the chitinase YlsC3 (WP_148025216.1), the glucanase YlsB3 (WP_148024776.1), and the mannosidase YlsA3 (WP_148025494.1). Degradation of laminarin and chitin biopolymers into monomer components could be demonstrated with YlsB3 and YlsC3, respectively, but no activity was detected for the mannosidase YlsA3. Collectively, our findings indicated that upon contact with yeast cells, *S. lavendulae* YAKB-15 produces an extensive set of extracellular CAZymes that can degrade key components of the yeast cell wall. The lack of enzymatic activity for YlsA3 against mannan may have been due to substrate specificity since the structural diversity of mannan polymers is diverse (43) and yeast-specific mannan is not commercially available.

### Metabolomic analysis suggests that yeast cell membrane components are utilized as nutrients by *S. lavendulae* YAKB-15

**Fig 3:**
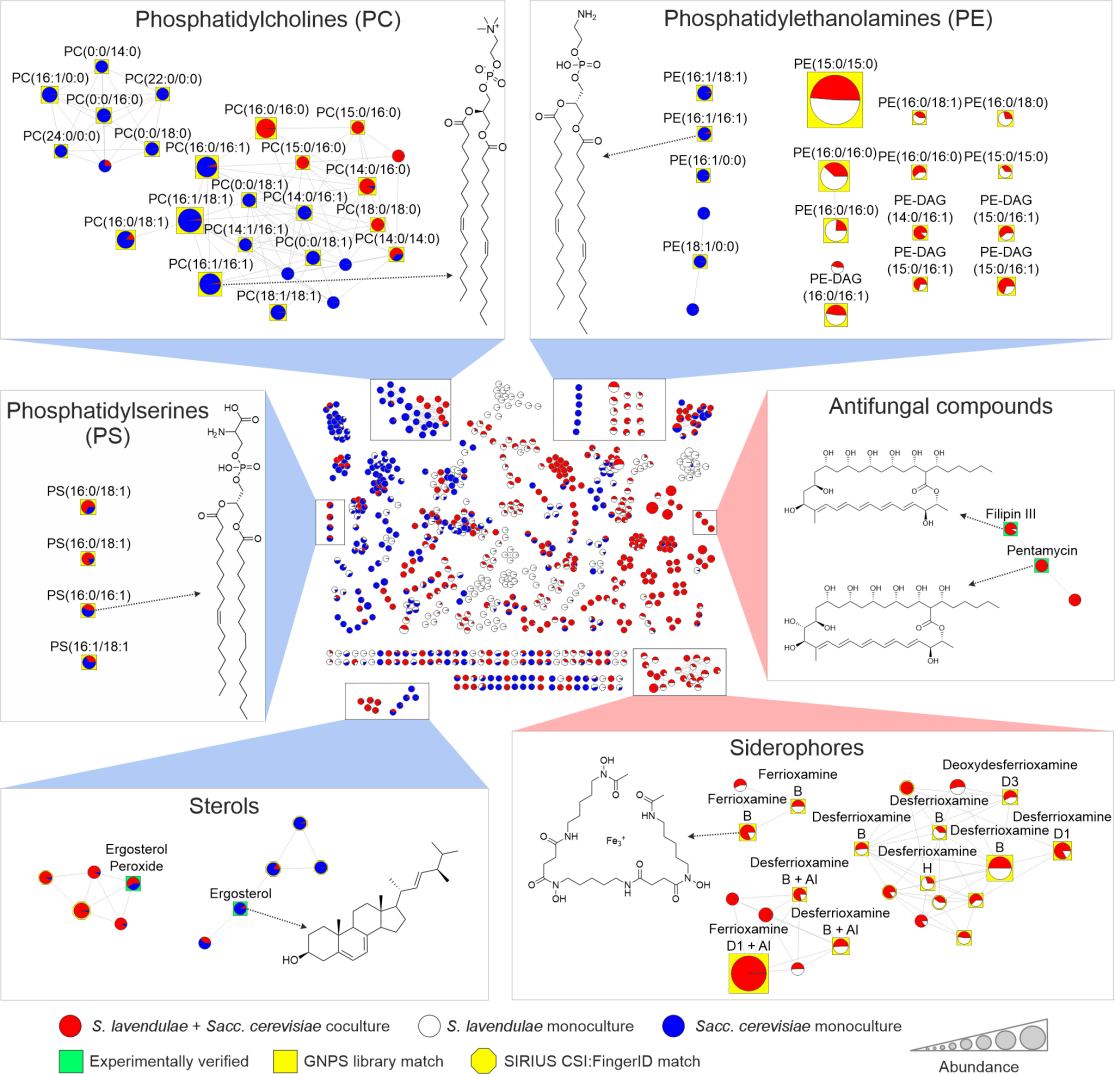
Feature-based molecular networking highlights metabolomic changes in *S. lavendulae* YAKB-15 during co-culture with live *Sacc. cerevisiae*. Under co-culture conditions (red), modified phosphatidylcholines (PCs) and phosphatidylethanolamines (PEs) and antifungal metabolites are detected that are not observed in axenic *Sacc. cerevisiae* (blue) or *S. lavendulae* YAKB-15 (white) cultures. Insets highlight the regions of interest in the molecular network. Node colors show the distribution of the parent ion intensity in different monoculture or coculture conditions. Identified nodes are labeled with the respective compound names. Node size indicates the sum of parent ion intensity in MS1 scans.

Having established that *S. lavendulae* secretes a suite of enzymes capable of dismantling the protective yeast cell wall, we next utilized Feature-based molecular networking (FBMN) (44) in the Global Natural Products Social Molecular Networking (GNPS) (45) infrastructure to investigate the subsequent assimilation of the exposed yeast cell membrane components by metabolomic analysis. To decipher the specific organism responsible for the observed metabolic changes, these experiments were conducted using both live yeast in SC medium (Fig. 3) and dead yeast in Y medium (Fig. S4).

We observed three key differences between axenic and co-culture samples with live yeast. First, yeast cell membrane components, including the primary phospholipids and sterols (46), disappeared within 5 days under co-culture conditions. *S. lavendulae* efficiently modified yeast-specific phosphatidylcholines (PCs) and phosphatidylethanolamines (PEs), and we observed biotransformation of a subset of PCs through the reduction of double bonds. Among annotated phosphatidylserines, PS (16:0/18:1) showed a marked increase in co-culture conditions (Fig. 3). Consistent with other membrane components, most ergosterol and ergosterol-like compounds were not present in co-culture samples, and several modified sterols not found in yeast monocultures appeared in the molecular network (Fig. 3). The result verified that *Streptomyces* are able to metabolize cell membrane components of live yeast cells. The metabolomic data correlated with the upregulation of sterol degrading enzymes and phospholipases observed in transcriptomic analysis.

The metabolomic analysis also confirmed the production of secondary metabolites under co-culture conditions. We detected the production of filipin-like antifungal polyenes, which target sterols in the yeast cell membrane, only under co-culture conditions (Fig. 3). Third, we observed upregulation in the production of ferrioxamine-type siderophores (9, 11) by *Streptomyces,* which is consistent with active competition for iron and potential signaling interactions between the two organisms.

Experiments with dead yeast cells (Fig. S4) correlated well with metabolomic data with live yeast cells (Fig. 3) with three notable exceptions. The appearance of ornithine lipids (OLs) was additionally detected in the co-culture with dead yeast (Fig. S4). OLs are phosphorus-free lipids commonly produced by bacteria under phosphate limitation or other stress conditions (47). In addition, siderophore production was downregulated under these conditions (Fig. S4), possibly due to availability of excess iron released from autoclaved yeast cells. Finally, we did not observe modified phosphatidylserines when autoclaved yeast was utilized, which was in contrast to the experiments with live yeast cells.

### *S. lavendulae* YAKB-15 produces pentamycin and cholesterol oxidase ChoD to destabilize the yeast cell membrane

**Fig 4:**
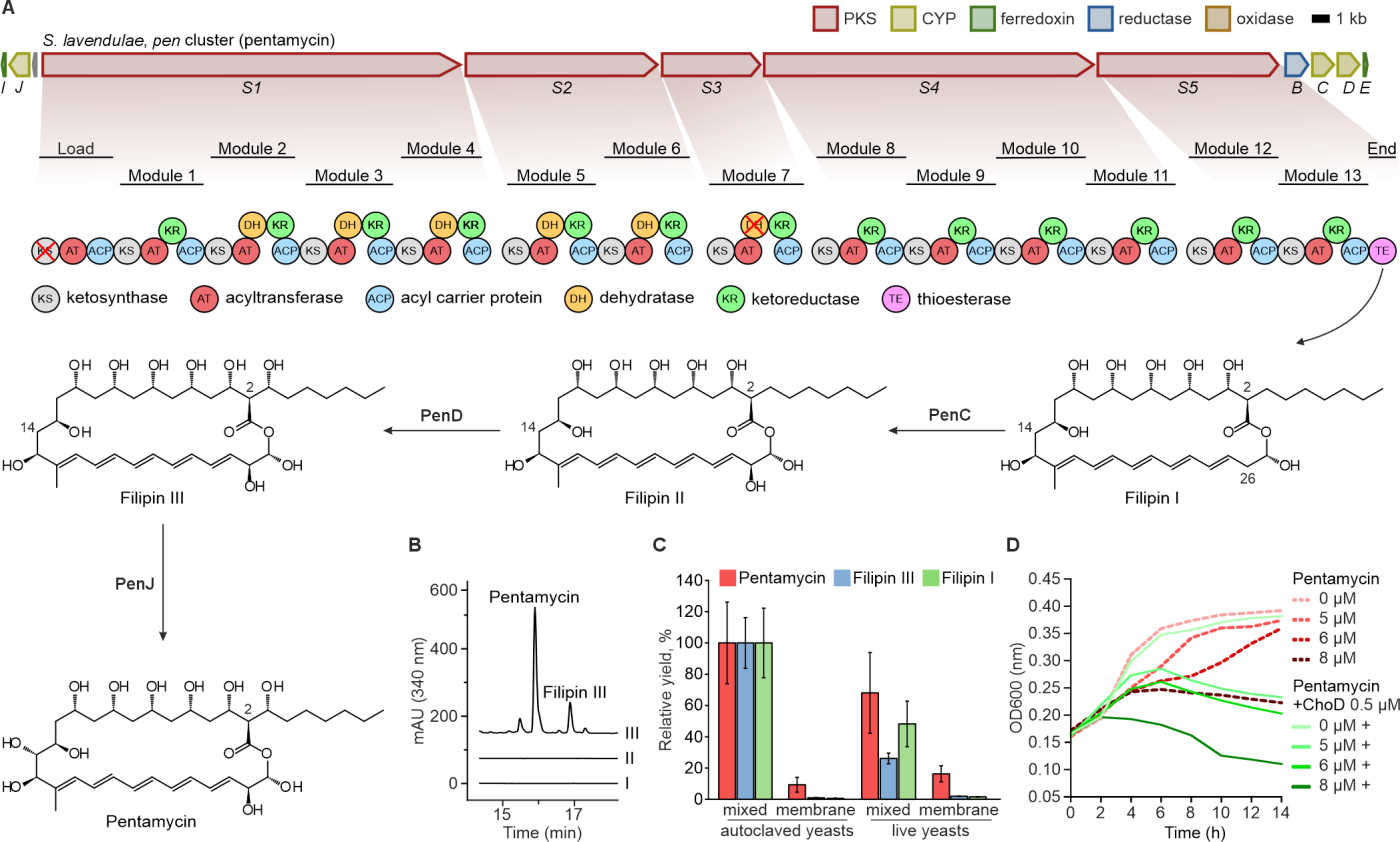
Production of polyenes by *S. lavendulae* YAKB-15 during interaction and synergistic bioactivity of pentamycin with cholesterol oxidase ChoD. (**A**) The pentamycin biosynthetic gene cluster (*pen*) and modular organization of the Type I polyketide synthase (PKS) domains. Essential domains (ketosynthase [KS], acyltransferase [AT], and acyl carrier protein [ACP]) drive elongation, while auxiliary ketoreductase (KR) and dehydratase (DH) domains determine the redox state. The thioesterase (TE) facilitates product release. Tailoring enzymes PenC, PenD, and PenJ sequentially convert filipin I to filipin III and pentamycin. (**B**) HPLC analysis of *S. lavendulae* culture extracts (340 nm). Traces: (I) axenic culture, (II) ∆PKS biosynthetic knockout co-culture with yeast, and (III) wild-type co-culture with yeast. (**C**) Relative yield of polyenes (pentamycin, filipin III, and filipin I). Production is stimulated by physical interaction but attenuated by a polyethylene terephthalate (PET) membrane. Values represent the mean ± SD (*n* = 3). (**D**) Yeast growth inhibition assays (OD_600_) demonstrating that ChoD (0.5 µM) enhances the antifungal bioactivity of pentamycin (0–8 µM).

**Fig 5:**
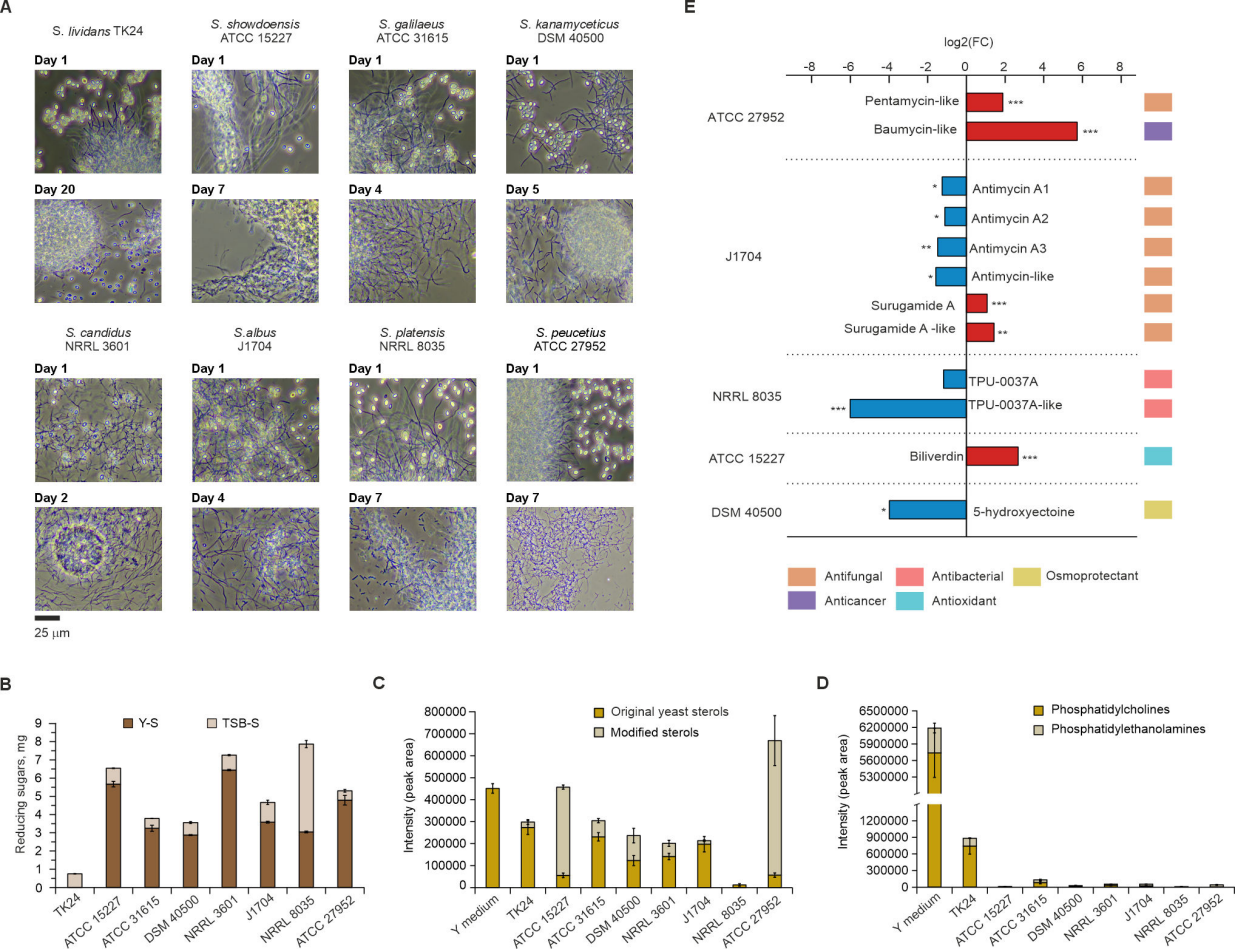
Yeast predation is a common phenomenon in *Streptomyces*. (**A**) Microscopic observations of eight *Streptomyces* strains during extended cultivations demonstrate predation by seven out of eight strains. (**B**) Enzymatic activity of the *Streptomyces* secretome from cultures with whole autoclaved yeast cells (Y-T) in comparison to cultures from yeast-free (TSB-T) media. Increased CAZyme activity is observed from co-cultures confirming degradation of yeast cell wall by seven out of eight strains when whole autoclaved yeast cells were used as substrates. Error bars indicate the standard deviation from three technical triplicates. (**C**) Degradation of yeast sterols based on metabolomic analyses. Chemically modified sterols were detected to varying extents from several strains. Error bar indicates standard deviation from three biological replicates. (**D**) Degradation of yeast lipids, phosphatidylcholines, and phosphatidylethanolamines based on metabolomic analyses. Seven out of eight *Streptomyces* strains appear to consume nearly all of the lipids. Error bar indicates standard deviation from three biological replicates. (**E**) Fold change analysis of secondary metabolites produced by *Streptomyces* strains under axenic and co-culture conditions based on metabolomic analyses. For panel E, means plus standard deviations (SDs) from three biological replicates are shown with statistical significance indicated as follows: ***, *P* < 0.01; **, *P* < 0.05; *, *P* < 0.1 with *t*-test.

The structures of the polyenes observed by transcriptomics and metabolomics were confirmed by 1D NMR (^1^H and ^13^C NMR) and 2D NMR (COSY, HSQC, and HMBC) (FigS5 through S17, Table S1 and S2). The compounds were isolated from large-scale co-cultures and were found to reside predominantly in the cell biomass fraction and not in the supernatant. NMR spectroscopy revealed that these co-culture exclusive natural products were the known polyenes pentamycin and filipin III (Fig. 4A). A type I polyketide synthase BGC with domain architecture suitable for production of the polyenes (Fig. 4A) was identified from the genome of *S. lavendulae* YAKB-15 due to the high (90.6%) average nucleotide sequence identity to a pentamycin BGC from *Streptomyces* sp. S816 (33). Inactivation of the *penS1* polyketide synthase gene led to cessation of polyene production in the mutant strain (Fig. 4B), which confirmed that the BGC is responsible for the biosynthesis of pentamycin and filipin III in *S. lavendulae* YAKB-15. It is noteworthy that the *pen* BGC did not encode any transporters and the polyenes were isolated from the cell biomass fractions, which indicated that the compounds were produced intracellularly. *Streptomyces* are known to produce lipid vesicles for delivery of secondary metabolites (48), and while this is challenging to prove unequivocally for *S. lavendulae* YAKB-15, it is interesting to note that a merger of such transport vesicles with the yeast cell membrane would result in the delivery of the polyenes to the vicinity of their biological target.

Next, we demonstrated that direct contact with yeast cells strongly influenced polyene biosynthesis in *S. lavandulae* YAKB-15. When *S. lavendulae* YAKB-15 was physically separated from *Sacc. cerevisiae* by a 0.4-µm membrane, the production of polyenes (including pentamycin, filipin III, and filipin I) was markedly reduced compared to direct co-cultures (Fig. 4C). This effect was observed with both live and autoclaved yeast, with membrane separation leading to decreases in polyene yield of over 75% and 90%, respectively.

Polyene antibiotics are effective antifungal agents that interact with sterols embedded in cell membranes. The interaction leads to cell death via sterol sequestering, membrane destabilization, or pore formation depending on the type of polyene (49). Because the cell-associated cholesterol oxidase ChoD targets the same membrane ergosterol and creates spatial structuring (50), we performed yeast inhibition assays to investigate possible cooperative bioactivity. *In vitro* susceptibility testing revealed that yeast growth was inhibited at 8 µM pentamycin concentration (Fig. 4C), which is in accordance with literature reports (51). However, in the presence of 0.5 µM ChoD, 5 µM pentamycin was sufficient to arrest yeast cell growth (Fig. 4C). While the molecular mechanism for the observed co-operativity requires further study, we propose that conversion of ergosterol to ergosta-4,7,22-trien-3-one (52) may facilitate sequestering of the sterol by pentamycin. This is supported by the observations that cholestenone desorbs more readily from membranes than cholesterol (53), and sterol extraction kinetics have been shown to influence the bioactivity of polyenes (54).

### Yeast predation is a common phenomenon in *Streptomyces*

Having characterized the multi-omics predatome of *S. lavendulae* YAKB-15, we next investigated whether yeast predation is a widespread phenomenon across the broader genus. To test this, we selected eight diverse *Streptomyces* species with sequenced genomes for co-culture experiments (Fig. 5A). Selected strains included six type strains *S. showdoensis* ATCC 15227, *S. galilaeus* ATCC 31615, *S. kanamyceticus* DSM 40500, *S. candidus* NRRL 3601, *S. platensis* NRRL 8035, and *S. peucetius* ATCC 27952 and two commonly used heterologous hosts *S. albus* J1704 and *S. lividans* TK24. Yeast cells disappeared in seven cases and only *S. lividans* TK24 appeared to be unable to digest yeast even after 20 days (Fig. 5A). The kinetics of predation varied, and yeast cells were assimilated in 2–7 days depending on the strain. Hydrolytic enzyme activity from culture supernatants was notably increased in six of the strains by 3.3- to 9.3-fold under co-culture conditions, while in the case of *S. platensis* NRRL 8035, the activity was reduced by 37% (Fig. 5B).

Metabolomic analysis of the eight *Streptomyces* strains with dead yeast cells revealed variation also in their abilities to degrade sterols and lipids from yeast cell membrane (Fig. 5C and D). *S. platensis* NRRL 8035 exhibited exceptional sterol degradation efficiency, nearly completely utilizing these compounds. In contrast, *S. lividans* TK24 displayed minimal alteration of the original sterol structure, maintaining up to 60.5% of the initial yeast sterols level (Fig. 5C). All strains demonstrated effective utilization of lipids, including phosphatidylcholines and phosphatidylethanolamines, from yeast membranes (Fig. 5D). The experiments suggested that the kinetics in yeast membrane destabilization differs greatly between various *Streptomyces* species.

The metabolomic data also revealed complex changes in the production of several natural products with antifungal, antibacterial, anticancer, antioxidant, and osmoprotective activities (Fig. 5E). When exposed to yeast, *S. peucetius* ATCC 27952 exhibited a twofold increase in the production of an unknown antifungal pentamycin-like polyene and a sixfold increase in the formation of anthracycline anticancer baumycin-like antibiotics, which the strain is known to produce (55). In *S. albus* J1704, we observed two antifungal natural products that the strain is known to produce (56, 57); production of suguramides was upregulated upon contact with yeast, but simultaneously antimycins were produced in lower quantities. It is also noteworthy that *S. albus* J1704 has been shown to harbor a candicidin-type polyene BGC (56), but we could not detect any polyenes from the strain. In the other three *Streptomyces* strains, we did not observe the production of any known antifungal metabolites. Upon contact with yeast, *S. platensis* NRRL 8035 downregulated the production of the lydicamycin-type antibiotic TPU-0037 (58), which has been shown to be effective against Gram-positive bacteria, while *S. showdoensis* ATCC 15227 initiated the production of the putative tetrapyrrole signaling molecule biliverdin (59) and *S. kanamyceticus* DSM 40500 downregulated the biosynthesis of the osmoprotectant 5-hydroxyectoine (60).

### Concluding remarks

**Fig 6:**
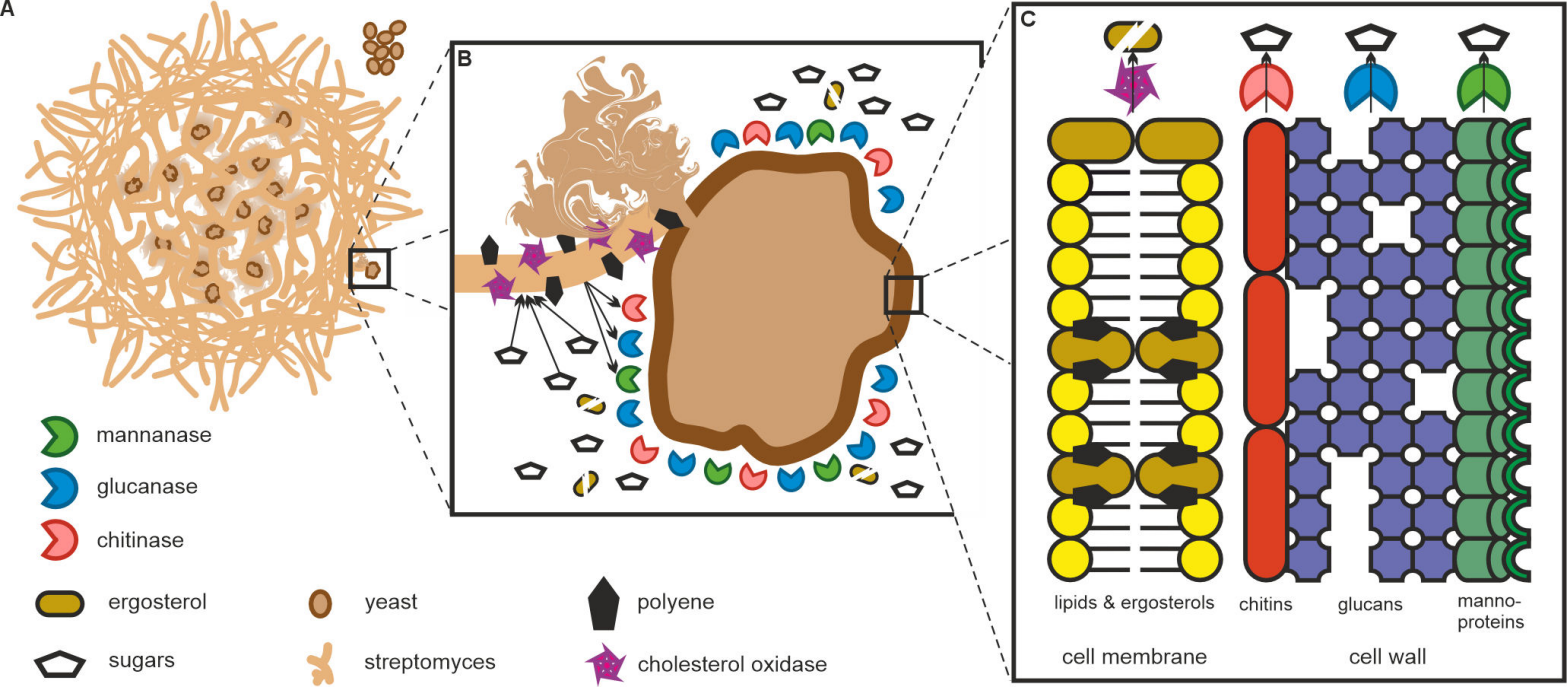
Model for predation of yeast by *Streptomyces*. (**A**) Physical contact triggers *Streptomyces* to attack yeast with multiple approaches. (**B**) *Streptomyces* produce cell-associated ChoD and the polyenes to destabilize yeast cell membranes and secreted CAZymes to digest the yeast cell wall (Fig. 2 to 5). (**C**) *Streptomyces* catabolize and use as nutrients key biological macromolecules of yeast. The mannoprotein, glucan, and chitin constituents of the yeast cell wall are degraded to individual carbohydrates by CAZymes. Membrane sterols are catabolized and yeast lipids may be converted to ornithine lipids by *Streptomyces*.

Microbial predation has been known to exist for several decades with *Bdellovibrio*, *Bradymonas*, and *Myxococcus* demonstrated to have diverse strategies for predation (61). Although *Streptomyces* are well-known producers of bioactive antibiotics and hydrolytic exoenzymes, they have not been classically considered predatory bacteria. Here, we show that several species of *Streptomyces* can assimilate yeast cells under co-cultures. Our model (Fig. 6) suggests that physical interactions trigger substantial changes in the transcriptome of both predator and prey. Our data suggest that *Streptomyces* species have widespread abilities to degrade major constituents of the yeast cell wall and membrane. We show that upon encountering yeast cells, *Streptomyces* produce CAZymes capable of digesting each component of the yeast cell wall composed of mannoprotein, glucan, and chitin layers. The upregulation of many predicted intracellular CAZymes (Fig. 2A) indicates that processing of the various carbohydrate biopolymers continues after cellular uptake by *Streptomyces*. We further demonstrate that the interaction induces the production of cholesterol oxidase ChoD and polyenes, which we show for the first time to synergistically disrupt the cell membrane *in vitro* (Fig. 6) at low micromolar polyene concentrations (Fig. 4C). The depletion of yeast cell membrane components such as sterols, sphingolipids, and fatty acids strongly suggests their assimilation as nutrients (Fig. 6), as supported by our metabolomics analyses (Fig. 3). Our combined data promote a predatory model where *Streptomyces* harness multiple strategies to attack yeast cells and is able to widely use various biological macromolecules and metabolites from yeast as nutrients.

Our transcriptomics and metabolomics data indicate that the role of natural products is highly complex in predation of yeast cells. The induction of production of known antifungal agents such as pentamycin upon contact with yeast is logical in *S. lavendulae* YAKB-15 (Fig. 4B), and it is noteworthy that genes responsible for the biosynthesis of other putative bioactive agents and various siderophores were both up- and downregulated (Fig. S2B). The same trend may be observed in the other studied *Streptomyces* strains (Fig. 5E), with metabolomics indicating the upregulation of polyene biosynthesis in *S. peucetius*, but both up- and downregulation of antifungal agents surugamide A and antimycins, respectively, in *S. albus*. The analyses are complicated by the multiple biological activities that a single natural product, such as antimycins (62), may harbor and the challenges of studying the ecological impacts of these bioactivities in the soil microbiome.

In conclusion, this study provides comprehensive evidence that *Streptomyces* can exhibit facultative predation on yeast. However, the systems biology approaches employed here were not sufficient to uncover the molecular mechanisms by which *Streptomyces* detect and target yeast cells. Future work should focus on identifying the proteins involved in mediating contact between *Streptomyces* mycelium and the yeast cell wall. Additionally, *Streptomyces* were long considered to be non-motile, which precludes the wolf-pack hunting strategies enabled by the gliding motility of *Myxococcus xanthus* (21). However, fungal interactions and volatile signaling molecules have recently been shown to trigger exploratory mobility in *Streptomyces* (63), which occurs at similar velocities as gliding motility in *M. xanthus* (64). Determining whether this exploratory behavior contributes to predatory activity represents an important next step. Finally, future research should explore the broader predatory potential of *Streptomyces*, including their ability to prey on other fungi and bacteria beyond *Sacc. cerevisiae*, and identify if a correlation exists between a specific prey species and specific catabolic enzymes and natural products.

## MATERIALS AND METHODS

### Microbial strains, plasmids, and culture conditions

Strains and plasmids used in this study are described in Table S3. *Streptomyces* cultures were generally grown at 30°C, shaking at 250–300 rpm. Strains of *Streptomyces* were cultivated in Y medium (9.1 g/L glucose, 2 g/L NH_4_NO_3_, 2 g/L CaCO_3_, and 26 g/L Baker’s yeast [26]) and in SC medium (0.2% [wt/vol] Synthetic Complete medium without specific amino acids, 1% [wt/vol] yeast nitrogen base supplemented with 2% [wt/vol] glucose) (65) with live yeast, and TSB (66) medium as described previously. The solid media used were YPD (65) and MS (66) as previously described.

### Microscopy

Confocal fluorescence microscopy was performed using a Nikon Eclipse Ti2-E microscope with a NikonDS-Fi3 CMOS camera and a Hamamatsu sCMOS Orca Flash 4 camera attached and a Lumencor Spectra X LED light source in a temperature-controlled incubation chamber. Images were acquired using a Nikon CFI S Plan Fluor EWLD 20×/0.45 DIC N1 objective with mCherry excitation/emission bandwidths of 555 nm/632 nm and GFP excitation/emission bandwidths of 488 nm/515 nm. Images were collected using NIS-Elements AR (Nikon) and analyzed using Fiji (67).

For time-lapse imaging of *S. lavendulae*/pS_GK_ChoD (68) (cholesterol oxidase promoter probe) and *Sacc. cerevisiae* constitutively expressing mCherry (27), strains were first grown in SC for 48 h. The co-cultures were initiated by mixing 100 µL of *Streptomyces* with 10 µL of yeast in 1 mL of SC in a six well plate. Each strain was also grown axenically using the same respective conditions. Experiments were performed at 30°C, and images were taken every 10 min for 24 h.

Standard microscopy images were captured using a Nikon Eclipse Ci-L upright microscope with a Canon EOS RP camera attached by a TUST38C LM Direct Image C-Mount Port (Micro Tech Lab, Graz, Austria) and a DSLRCRFTC_Pro LM Digital SLR Universal Adapter (Micro Tech Lab, Graz, Austria).

For scanning electron microscopy, *S. lavendulae* and *Sacc. cerevisiae* were co-cultured in SC medium for 3 days at 30°C. Axenic cultures of each strain were prepared under identical conditions. Cells were harvested by centrifugation and fixed in 1 mL of 2.5% glutaraldehyde at room temperature. Subsequent sample preparation followed the hexamethyldisilazane (HMDS) dehydration method. The specimens were then imaged using an Apreo S field-emission scanning electron microscope (Thermo Scientific).

### Chemical analysis

Compounds were extracted with methanol from the cell mass. Methanol extract was evaporated using a rotary evaporator, and compounds were resuspended in H_2_O. Polyenes were extracted from aqueous phase with ethyl acetate, and ethyl acetate phase was dried using a rotary evaporator. Polyenes were further purified using first silica chromatography followed by semi-preparative HPLC. Fractions containing pure compounds were extracted with ethyl acetate, dried using rotary evaporator and desiccator, and resuspended in deuterated solvents (Eurisotop) for NMR measurements.

NMR spectra were recorded with 600 MHz Bruker AVANCE-III system with liquid nitrogen cooled Prodigy TCI cryoprobe or 500 MHz Bruker AVANCE-III system with liquid nitrogen cooled Prodigy BBO cryoprobe. All NMR spectra were processed in Bruker TopSpin 4.1.3 version, and the signals were internally referenced to the solvent signals or tetramethylsilane. High-resolution electrospray ionization mass spectra were recorded on Bruker Daltonics micrOTOF system.

HPLC-UV analyses were carried out using a SCL-10Avp/SpdM10Avp system with a diode array detector (Shimadzu) and a C18 column (2.6 μm, 100 Å, 4.6 × 100 mm Kinetex column [Phenomenex]). HPLC-UV method: solvent A: 0.1% formic acid, 15% CH_3_CN, 85% H_2_O; solvent B: 100% CH_3_CN; flow rate: 0.5 mL/min; 0–2 min, 0% B; 2–20 min, 0%–60% B; 20–24 min, 100% B; 24–29 min, 0% B. HPLC-MS analyses were carried out using an Agilent 6120 Quadrupole LCMS system linked to an Agilent Technologies 1260 infinity HPLC system using identical columns, gradients, and buffers as for HPLC-UV analyses.

Semi-preparative HPLC were carried out using a LC-20AP/CBM-20A system with a diode array detector (Shimadzu) and EVO C18, 5 μm, 100 Å, 250 × 21.2 mm Kinetex column (Phenomenex). Semi-preparative HPLC method: solvent A: 50% 60 mM ammonium acetate–acetic acid pH 3.6, 15% CH_3_CN, 35% H_2_O; solvent B: CH_3_CN; flowrate: 20 mL/min; 0–2 min, 0% B; 2–20 min, 0%–60% B; 20–24 min, 100% B; 24–29 min, 0% B. Silica chromatography was performed using high-purity grade silica (pore size 60 Å, 230–400 mesh particle size) and a gradient elution from 100:0 CHCl_3_/MeOH to 0:100 CHCl_3_/MeOH. All reagents were purchased from Sigma-Aldrich unless stated otherwise.

### Bioinformatics

The accession number of the *S. lavendulae* YAKB-15 genome is GCA_008016805.1, and for the *Sacc. cerevisiae* genome, the accession number is GCA_000146045.2. BGCs were identified using antiSMASH v6.1.1 (36). PKS genes were analyzed using SeMPI 2.0 (69). CAZymes were functionally annotated using dbCAN2 (70). The average nucleotide identity was calculated using OrthoANIu (71).

### Transcriptomics

Cultures for time-resolved transcriptomal profiling were performed in biological quadruplets at 30°C and 300 rpm. For the live yeast cultures, SC was used and samples were taken on days 1, 3, 5, and 7. For the dead yeast cultures, Y medium was used and samples were taken at 6, 12, 24, and 48 h. Axenic cultures were grown in the same respective medium without yeast. To ensure the biological relevance of transcriptomic shifts observed in pooled samples, we independently validated key upregulated pathways, specifically the CAZymes and polyene biosynthesis, through downstream proteomics, metabolomics, specific enzyme activity assays and quantitative chromatographic detection.

RNA extraction was performed by pooling 1 mL of four independent cultures and adding 444 µL of cold STOP solution (5% phenol in ethanol). The samples were pelleted by centrifugation (5,000 × *g*, 10 min, 4°C), flash-frozen, and kept at −80°C (maximum 2 months). The cells were lysed with a mortar and pestle under liquid nitrogen, and then, RNA was isolated using an RNeasy Mini Kit (Qiagen) with DNase treatment. Total RNA was sent to Novogene (Cambridge, UK) for quality control (Agilent 2100), rRNA depletion (Ribo-Zero kit), library preparation (Illumina), and sequencing with NovaSeq 6000 (Illumina) to produce 2 × 150 bp reads.

All analyses were performed using the Chipster (72) platform. The reads were manually checked using FASTQC (73), and trimming was performed using TRIMMOMATIC (74). The trimmed reads were aligned to the genome using BowTie2 (75) and counted using HTSeq (76). Differential expression was performed using edgeR (77). Active BGCs were determined as previously described (78), with an average transcripts per million (TPM) value of 90 as established by the detection of pentamycin.

### Proteomics

Proteomics analysis was performed on cut pieces of SDS-PAGE gel (~55 kDa) from the supernatant of *S. lavendulae* cultured for 24 h under the same conditions as the transcriptomics experiments. In-gel digestion was performed at the Turku Proteomics Facility (Turku, Finland) according to standard protocol. The samples were analyzed by LC-ESI-MS/MS using a nanoflow HPLC (Thermo Fisher) coupled to a Q Exactive HF mass spectrometer (Thermo Fisher) equipped with a nano-electrospray ionization source. Peptides were resolved on a trapping column and subsequently separated inline on a 15 cm C18 column (75 μm × 15 cm, ReproSil-Pur 3 μm 120 Å C18-AQ, Dr. Maisch HPLC GmbH, Ammerbuch-Entringen, Germany). Peptides were eluted with a 20 min gradient of 6%–39%, followed by a 10 min wash stage with 100% at acetonitrile/water (80:20 [vol/vol]) with 0.1% formic acid.

MS data were acquired using Thermo Xcalibur v4.1 (Thermo Fisher). An information dependent acquisition method consisted of an Orbitrap MS survey scan of mass range 350–1,750 *m/z* followed by HCD fragmentation for the 10 most intense peptide ions. Protein identification searches were performed using Proteome Discoverer v2.5 (Thermo Fisher) connected to an in-house server running Mascot v2.7.0 (Matrix Science). Data were searched against a Swissprot *Streptomyces* database (downloaded 18.10.2021). The database search parameters were trypsin for the enzyme, and two missed cleavages were allowed. Cysteine carbamidomethylation was set as static modification, and methionine oxidation and protein N-terminal acetylation were set as variable modifications. The peptide mass tolerance was ±10 ppm, and the fragment mass tolerance was ±0.02 Da.

### Protein production

YlsC3, YlsB3, and YlsA3 were heterologously produced in *Escherichia coli* TOP10 strain transformed with pBADHisBΔ-ylsC3, pBADHisBΔ-ylsB3, pBADHisBΔ-ylsA3, respectively. The pre-culture was grown overnight in LB medium with 100 µg/mL of ampicillin and inoculated (1%) in 4 × 500 mL of 2xTY medium with 100 µg/mL of ampicillin. After incubation (30°C, 4 h, 250 rpm) to an OD_600_ of 0.6, cells were induced with 0.02% (wt/vol) ʟ-arabinose and incubated further overnight at room temperature at 180 rpm. Cultures were centrifuged (12,000 × *g*, 25 min, 4°C), and cell pellet was resuspended in 3 volumes of wash buffer (K_2_HPO_4_ 50 mM, imidazole 5 mM, NaCl 50 mM, 10% glycerol, 1% Triton X) and subsequently sonicated (Soniprep 150, MSE). Samples were centrifuged (18,000 × *g*, 30 min, 4°C), and the supernatant was collected and mixed with TALON Superflow affinity resin (GE Healthcare). After incubation for 1 h at 4°C, the resin was washed with wash buffer and protein was eluted with 2.5 mL elution buffer (K_2_HPO_4_ 50 mM, imidazole 250 mM, NaCl 50 mM, 10% glycerol). Then, the sample was buffer exchanged to the storage buffer (K_2_HPO_4_ 50 mM, NaCl 50 mM, 10% glycerol) using a PD-10-column (GE Healthcare). The purified enzymes were analyzed by SDS-PAGE and reducing sugars assay. *E. coli* TOP10 transformed with an empty pBADHisBΔ (79) vector was used as a negative control for recombinant enzymes production and enzymatic assays.

### Enzymatic assays

To prepare culture supernatant for enzymatic assay, *Streptomyces* strains were cultured with autoclaved yeast (Y medium) and without (TSB medium) for 3 days. The supernatants were collected from centrifuged cultures (4,000 × *g*, 10 min, 4°C) and filtered through 0.45-μm syringe filters, followed by concentration (fivefold) in Amicon Ultra-15 centrifugal filters (Millipore, 10000 MWCO). Concentrated supernatants were stored at 4°C.

DNS (3,5-dinitrosalicylic acid) assay (80) was performed to measure enzymatic activities of purified enzymes and concentrated supernatants with laminarin, colloidal chitin, mannan, and 25% (wt/vol) yeast cells solution as substrates. The assay was performed as follows: 50 µL of chitinase (15 μM) or glucanase (23 μM) or mannosidase (7 μM) or culture supernatant was mixed with 450 µL of the substrate (10 mg/mL in 50 mM phosphate buffer, pH 7) and incubated at 37°C overnight. The reaction was stopped by adding 750 µL of DNS reagent and incubating samples at 95°C for 10 min. Samples were centrifuged (12,000 × *g*, 10 min) and subjected to absorbance reading at 540 nm. Substrate sample without enzyme was used as a reaction control. Concentration (mg/mL) of reducing sugars in the sample was calculated based on glucose standard curve.

### Metabolomics sample preparation, data collection, and Processing

Cultures for metabolomics samples were grown as three independent biological replicates for each strain. To identify metabolites induced by co-culture and determine their origin, we compared *S. lavendulae* YAKB-15 grown with live yeast to monocultures and to controls grown with dead yeast in Y-medium. Metabolites that appear in co-culture, but are absent in monocultures, are considered co-culture-induced. Since dead yeast are metabolically inactive, compounds that are formed during the time course experiments in both live-yeast and dead-yeast co-cultures can be likely attributed to the metabolic activity of *S. lavendulae* YAKB-15 rather than yeast, which has allowed us to distinguish yeast-derived metabolites from those produced or modified by the bacterium. For the live yeast experiments, *S. lavendulae* YAKB-15 and *Sacc. cerevisiae* were co-cultured in SC medium for 5 days at 30°C. For the dead yeast experiments, in both Y medium and SC medium. Cultures were incubated at 30°C and 300 rpm for 4 days. Axenic cultures of each strain were prepared under identical conditions as controls. For extraction, 20 mL of each culture was mixed with 6 mL of chloroform: methanol (2:1, vol/vol) containing internal standards (5 ppm caffeine, 5 ppm reserpine). Samples were sonicated for 5 min and vortexed for 30 min. Methanol and chloroform phases were collected separately, and the chloroform phase was dried in a vacuum concentrator and resuspended in 2 mL of methanol. Samples were filtered using Protein Precipitation Filter 96-well solid phase extraction (SPE) Plate (Supelco). Mass spectrometry was carried out using Sciex ExionLC UPLC system coupled to Sciex TripleTOF 6600 mass spectrometer and Atlantis Premier BEH C18 AX column, 1.7 µm, 2.1 × 100 mm (Waters). Method: solvent A: 0.1% formic acid, 10 mM ammonium formate in H2O; solvent B: 0.1% formic acid, 10 mM ammonium formate in MeOH; flow rate: 0.4 mL/min; 0–2 min, 0% B; 2–4 min, 0%–100% B; 4–10 min, 100% B; 10–16 min, 0% B. The mass spectrometry data were processed with MZmine3 (81), and the results were exported to GNPS for FBMN analysis.

### Molecular network analysis

A molecular network was created with the Feature-Based Molecular Networking (FBMN) (44) workflow on GNPS (45) (https://gnps.ucsd.edu). The data were filtered by removing all MS/MS fragment ions within ±17 Da of the precursor *m*/*z*. MS/MS spectra were window filtered by choosing only the top six fragment ions in the ±50 Da window throughout the spectrum. The precursor ion mass tolerance was set to 0.02 Da and the MS/MS fragment ion tolerance to 0.02 Da. A molecular network was then created where edges were filtered to have a cosine score above 0.75 and more than six matched peaks. Furthermore, edges between two nodes were kept in the network if and only if each of the nodes appeared in each other’s respective top 10 most similar nodes. Finally, the maximum size of a molecular family was set to 50, and the lowest-scoring edges were removed from molecular families until the molecular family size was below this threshold. The spectra in the network were then searched against GNPS spectral libraries (45, 82). The library spectra were filtered in the same manner as the input data. All matches kept between network spectra, and library spectra were required to have a score above 0.7 and at least six matched peaks. The molecular networks were visualized using Cytoscape 3.10.2 software (83).

Key metabolites were identified with either level 1 confidence (NMR characterization or authentic standard) or level 2 confidence (hit in GNPS library). Additionally, molecular network annotations were improved using Sirius 5.8.6 (84) and CSI:FingerID (85) structural prediction tool. CSI:FingerID score −100 was used as a cut-off value for the candidate structures, and the fragmentation patterns of the candidate structures were assessed manually. Quantitative values obtained through GNPS Feature-Based Molecular Networking represent relative quantification rather than absolute concentrations. Feature intensities were derived from LC–MS peak areas following feature detection and alignment in MZmine3 and exported as a feature quantification table for FBMN analysis. These values reflect the relative abundance of molecular features across samples, as described previously (44).

### Statistical analysis

Statistical analysis was performed with MetaboAnalyst 6.0 (86). Significantly upregulated metabolites were determined by having fold change (FC) threshold of 2 and adjusted *P*-value (FDR) threshold of 0.05 in *t*-test. All samples had three independent biological replicates. All data are presented as mean ± s.d.

### Yeast inhibition bioassay

The antifungal activity of pentamycin was evaluated using a liquid growth inhibition assay in a 96-well plate format. A 4-day-old preculture of *Sacc. cerevisiae* grown in synthetic complete (SC) media was diluted to a starting optical density (OD₆₀₀) of 0.15–0.2. Each well was inoculated with 100 µL of this yeast suspension. To assess its direct effect, pentamycin was added to achieve final concentrations of 0, 5, 6, and 8 µM. For combination experiments, cholesterol oxidase (ChoD) was added to a final concentration of 0.5 µM in conjunction with the same varying concentrations of pentamycin. The final volume in all wells was adjusted to 300 µL with fresh SC media. The plates were incubated at an appropriate temperature, and yeast growth was monitored by measuring the OD₆₀₀ every 2 h over 24 h using a Multiskan GO plate reader.

### Physically separated co-culture

Co-culture experiments were conducted using sterile cellQART 6-well plates equipped with translucent PET membrane inserts (0.4 µm pore size). For physically separated co-cultures with live yeast, the outer compartment (well) was filled with 3.5 mL of SC medium and inoculated with 30 µL of *Saccharomyces cerevisiae* culture. The inner compartment (insert) was supplied with 2.2 mL of SC medium and inoculated with 300 µL of *Streptomyces lavendulae* YAKB-15 pre-culture. As a mixed-culture control, 300 µL of *S. lavendulae* YAKB-15 and 30 µL of *Sacc. cerevisiae* were co-inoculated in standard 6-well plates containing 5.6 mL of SC medium. For experiments using autoclaved yeast, the outer compartment was filled with 3.5 mL of Y medium containing autoclaved yeast. The inner compartment was filled with 2.2 mL of Y medium without yeast and inoculated with 300 µL of *S. lavendulae* pre-culture. The corresponding control consisted of 300 µL of *S. lavendulae* grown in standard 6-well plates containing 5.6 mL of Y medium supplemented with autoclaved yeast. All plates were incubated at 30°C for 3 days with shaking at 100 rpm. For relative quantification of polyene production, the average yield from the direct co-culture with autoclaved yeast was set as the 100% reference.

### Construction of *S. Lavendulae* mutant

To establish a direct link between the *pen* biosynthetic gene cluster and polyene production, a targeted deletion of the polyketide synthase gene *penS1* was generated in *S. lavendulae* YAKB-15 via homologous recombination using the unstable multicopy pWHM3-based vector (87), pWHM3-oriT (provided by Prof. Gilles van Wezel, Leiden University, The Netherlands). First, 1 kb flanking regions upstream and downstream of the target *penS1* gene were synthesized. To facilitate selection, the apramycin resistance gene *aac (3)IV* was cloned between the homology flanks utilizing SpeI and BclI restriction sites. Each disruption cassette was subsequently cloned into the HindIII and XbaI sites of the pWHM3-oriT vector to create the final knockout plasmid.

Gene disruptions were generated by transferring the disruption construct into the *Escherichia coli* conjugation donor strain ET12567/pUZ8002 and performing intergeneric conjugation with the *S. lavendulae* YAKB-15 wild-type strain. To isolate double-crossover recombinants, the resulting exconjugants were subjected to several passages under non-selective conditions on MS plates. The successful ΔPKS double-crossover mutants were then obtained following further screening based on their expected antibiotic resistance profile: the acquisition of apramycin resistance (indicating integration of the cassette) and the simultaneous loss of thiostrepton resistance (indicating the loss of the vector backbone).

### Yeast viability and colony-forming unit assays

*S. lavendulae* YAKB-15 was cultivated either axenically or co-cultured with *Saccharomyces cerevisiae*. Axenic yeast cultures were included as a control. Cultures were incubated at 30°C shaking at 300 rpm in SC medium, and samples were collected daily over 5 days. At each time point, culture samples were serially diluted in 0.9% (wt/vol) NaCl supplemented with 0.1% (vol/vol) Tween 80 to minimize aggregation and improve dispersion of mycelial fragments and sequestered yeast. Dilutions were plated on SC solid medium supplemented with 50 μg/mL thiostrepton to select for yeast. Colony-forming unit (CFU) values are reported as Log_10_CFU/mL and determined after at least 2 days of incubation. Mean CFU and standard deviations were calculated from biological triplicates for each condition and time point.

## Data Availability

The RNA-seq transcriptomics data generated in this study have been deposited in the NCBI Gene Expression Omnibus (GEO) database under the accession number GSE228628. All mass spectrometry data are publicly available at the MassIVE data repository (https://massive.ucsd.edu/) with the following accession number: MSV000095781. The MS2 spectra of the metabolites identified via NMR have been deposited to the GNPS library. The GNPS jobs are available at the following links: https://gnps.ucsd.edu/ProteoSAFe/status.jsp?task=9bef531715044341813f1d6d2e10fb3b https://gnps.ucsd.edu/ProteoSAFe/status.jsp?task=b030ba536772449e80165d3de5ba48ce https://gnps.ucsd.edu/ProteoSAFe/status.jsp?task=7992b630ec4c422ca1a65fae5dfcc606 https://gnps.ucsd.edu/ProteoSAFe/status.jsp?task=b8282a9ed3de44f1beed797094e1ccd6 https://gnps.ucsd.edu/ProteoSAFe/status.jsp?task=8b296ea127a345e5bbe0a3630c0b3378 https://gnps.ucsd.edu/ProteoSAFe/status.jsp?task=16b5d52acc564f079e63610df8fccbd2 https://gnps.ucsd.edu/ProteoSAFe/status.jsp?task=da9e1e25408d45edbd0e1e34edfb2185 https://gnps.ucsd.edu/ProteoSAFe/status.jsp?task=d5c3ca9bacf444e3ac54432212df028f https://gnps.ucsd.edu/ProteoSAFe/status.jsp?task=286b22365e3443c1aff3f2a7d7ca9478 https://gnps.ucsd.edu/ProteoSAFe/status.jsp?task=dabc838bb1424ed0bad03d6135589648.

## SUPPLEMENTAL MATERIAL

Data S1 mbio.00563-26-s0001.xlsx Proteomics data.

Supplemental material mbio.00563-26-s0002.pdf Supplemental figures and tables; legends for Data S1 and supplemental movies.

Movie S1 mbio.00563-26-s0003.mp4 Confocal microscopy.

Movie S2 mbio.00563-26-s0004.mp4 Confocal microscopy closeup.

ASM does not own the copyrights to Supplemental Material that may be linked to, or accessed through, an article. The authors have granted ASM a non-exclusive, world-wide license to publish the Supplemental Material files. Please contact the corresponding author directly for reuse.
