# Supplementary material for: Facultative predation expands the ecological repertoire of Streptomyces
Source: mBio. 2026 Apr 20;17(5):e00563-26. doi: 10.1128/mbio.00563-26 (PMC13170338; doi:10.1128/mbio.00563-26)
Supplement: Supplemental material — Supplemental figures and tables; legends for Data S1 and supplemental movies. [file mbio.00563-26-s0002.pdf]

Supplementary Materials for

Facultative predation expands the ecological repertoire of *Streptomyces*

Keith Yamada, Arina Koroleva, Heli Tirkkonen, Vilja Siitonen, Mitchell Laughlin, Amanda Moglia, Soheila Matroodi, Amir Akhgari, Guillaume Mazurier, Jarmo Niemi, Mikko Metsä-Ketelä*

*Corresponding author: Mikko Metsä-Ketelä, mianme@utu.fi

This PDF file includes:

Figs. S1 to S17

Tables S1 to S3

Legends for Movies S1 to S2

Legend for Data S1

Other Supplementary Materials for this manuscript include the following:

Movies S1 to S2

Data S1

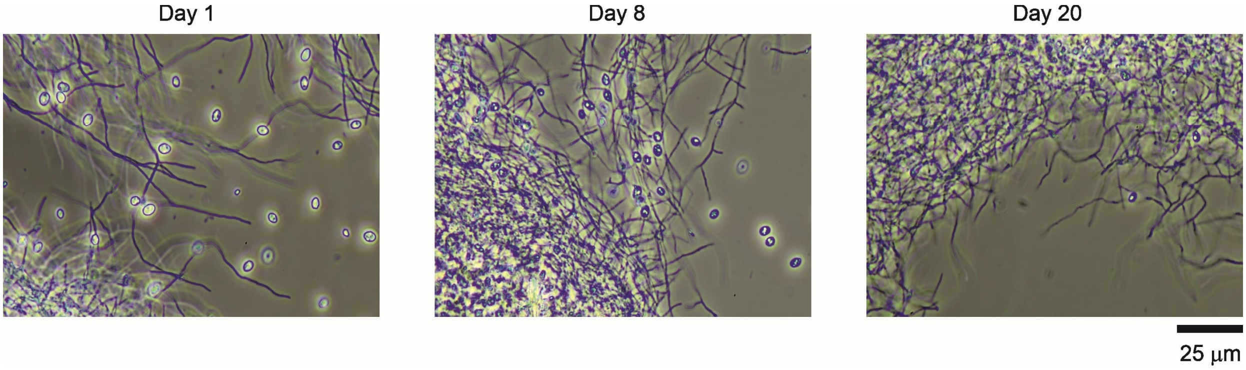

Fig. S1. Initial microscopic observation. Complete disappearance of autoclaved yeast cells from *Streptomyces lavendulae* YAKB-15 culture after 20 days.

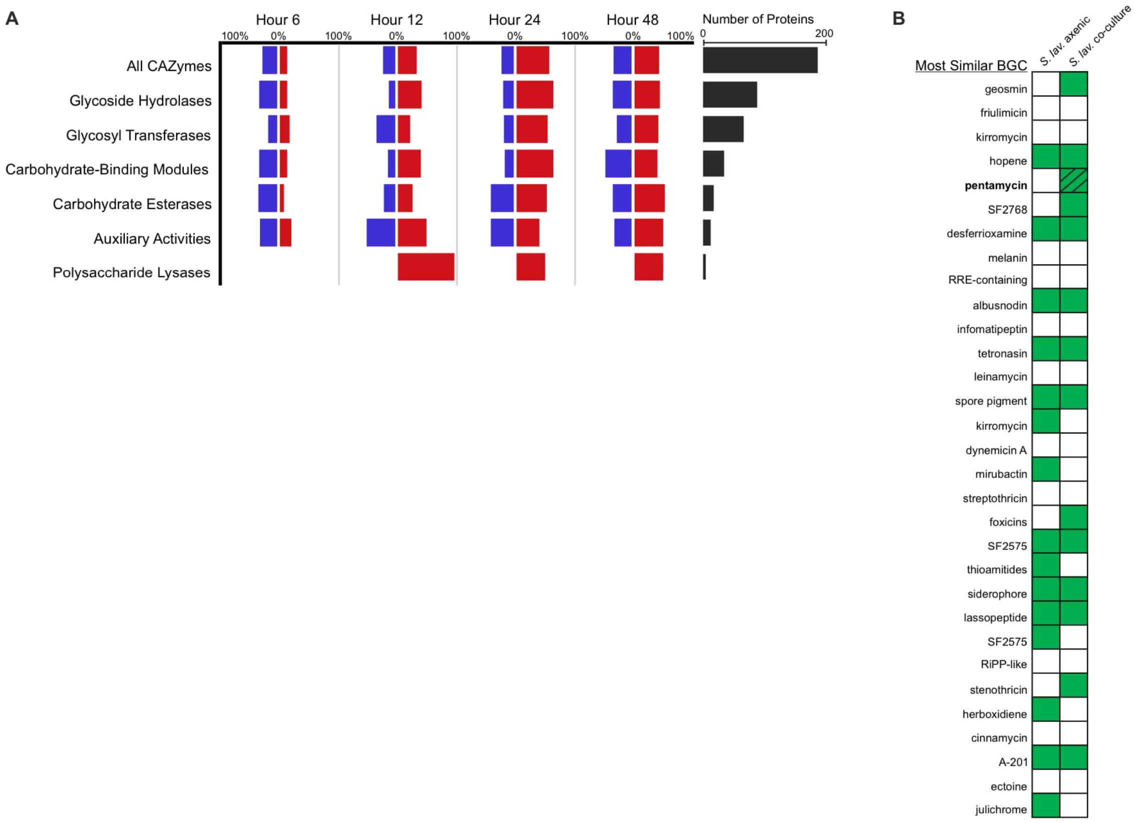

Fig. S2. Comparative transcriptomics profiling of CAZymes and BGCs. (A) Percentage of CAZymes up- or downregulated and the total number of CAZymes. *S. lavendulae* YAKB-15 CAZymes were evaluated between axenic and dead-yeast co-cultures across a time course of 6, 12, 24, and 48 hours. The total number of proteins with changes in gene transcription is presented in black on the x-axis for each category. Upregulated CAZymes are indicated in red and downregulated CAZymes are indicated in blue as percentage of the total number of proteins. The majority of the CAZymes are upregulated after 12 h. (B) Active *S. lavendulae* BGCs at 24 hours when axenically cultured or co-cultured with live yeast. Active BGCs have an average TPM count above 90 are shown in green. Identified compounds are shaded.

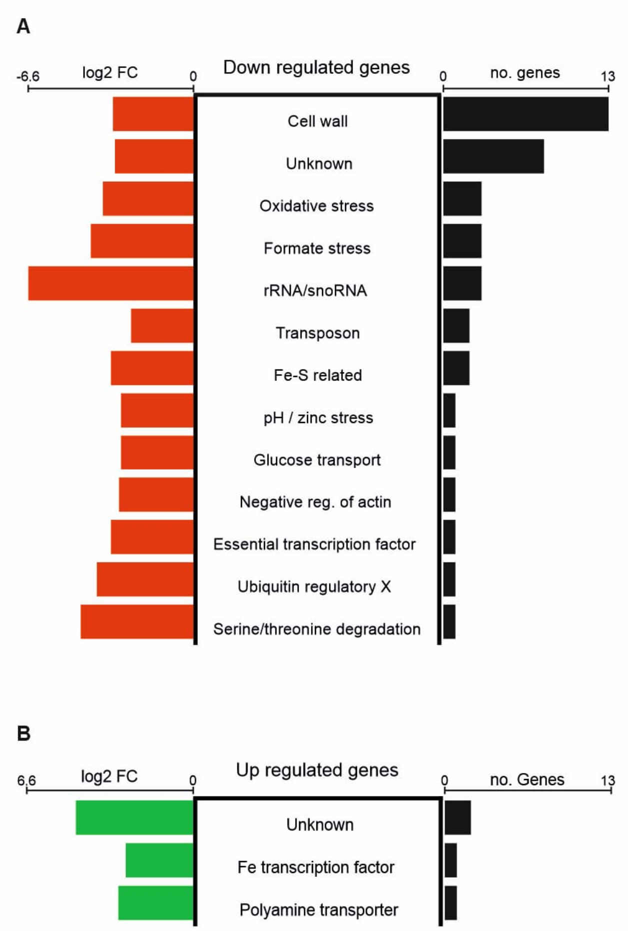

Fig. S3. Transcriptome analysis of *Sacc. cerevisiae* BY25610 co-cultured with *S. lavendulae.* (A) Downregulated genes and their function, differential expression, and number. (B) Upregulated genes and their function, differential expression, and number.

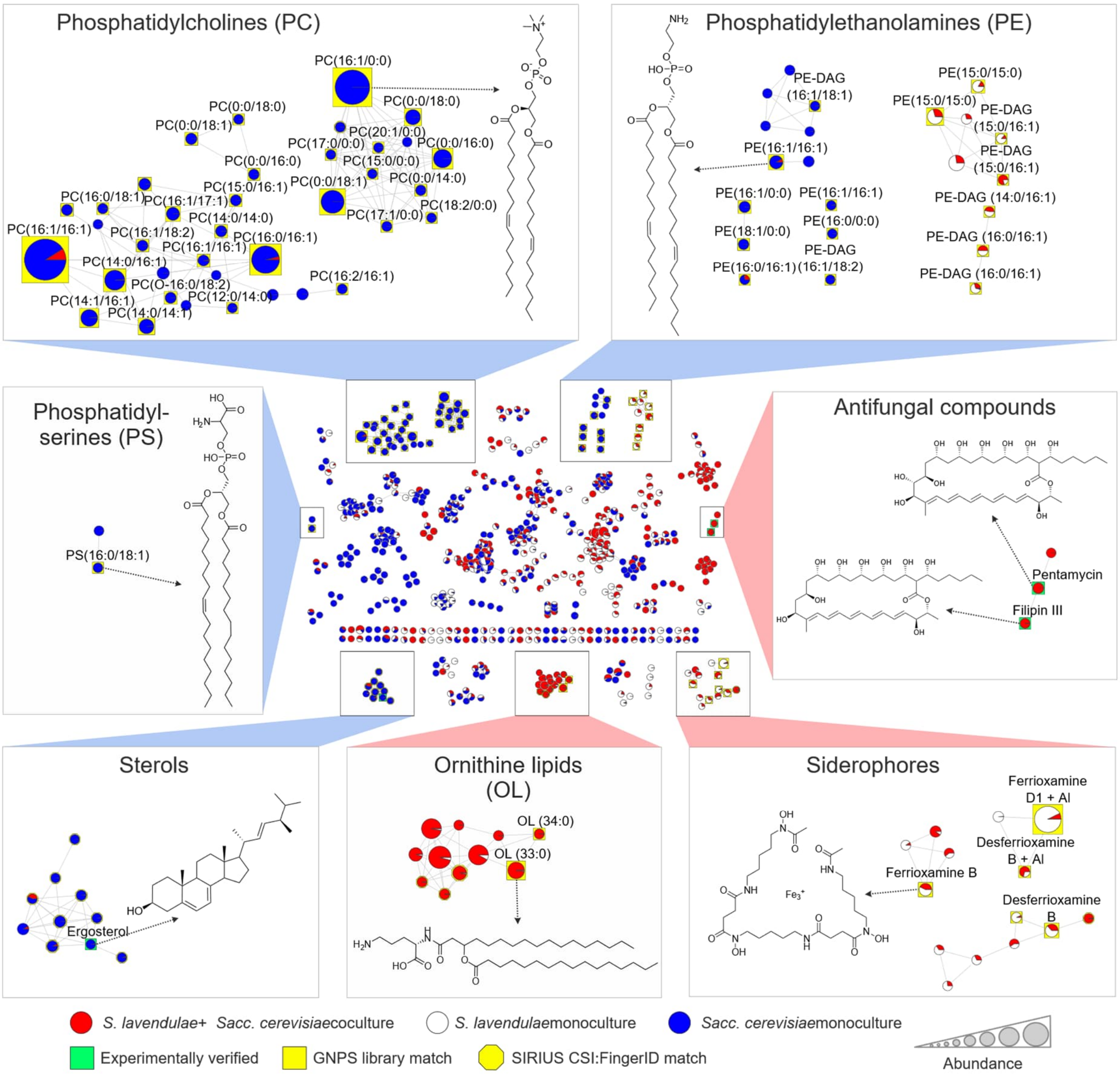

Fig. S4. Feature-based molecular network highlights the changes in *S. lavendulae* metabolome in the medium supplemented with autoclaved yeasts. *S. lavendulae* appears to have consumed the PCs and PEs present in the original yeast cell mass, whereas ornithine lipids and antifungal compounds are produced in the co-culture conditions. Insets highlight the regions of interest in the molecular network. Node colors show the distribution of the parent ion intensity in different monoculture or coculture conditions. Identified nodes are labeled with parent ion m/z. Node size indicates the sum of parent ion intensity in MS1 scans.

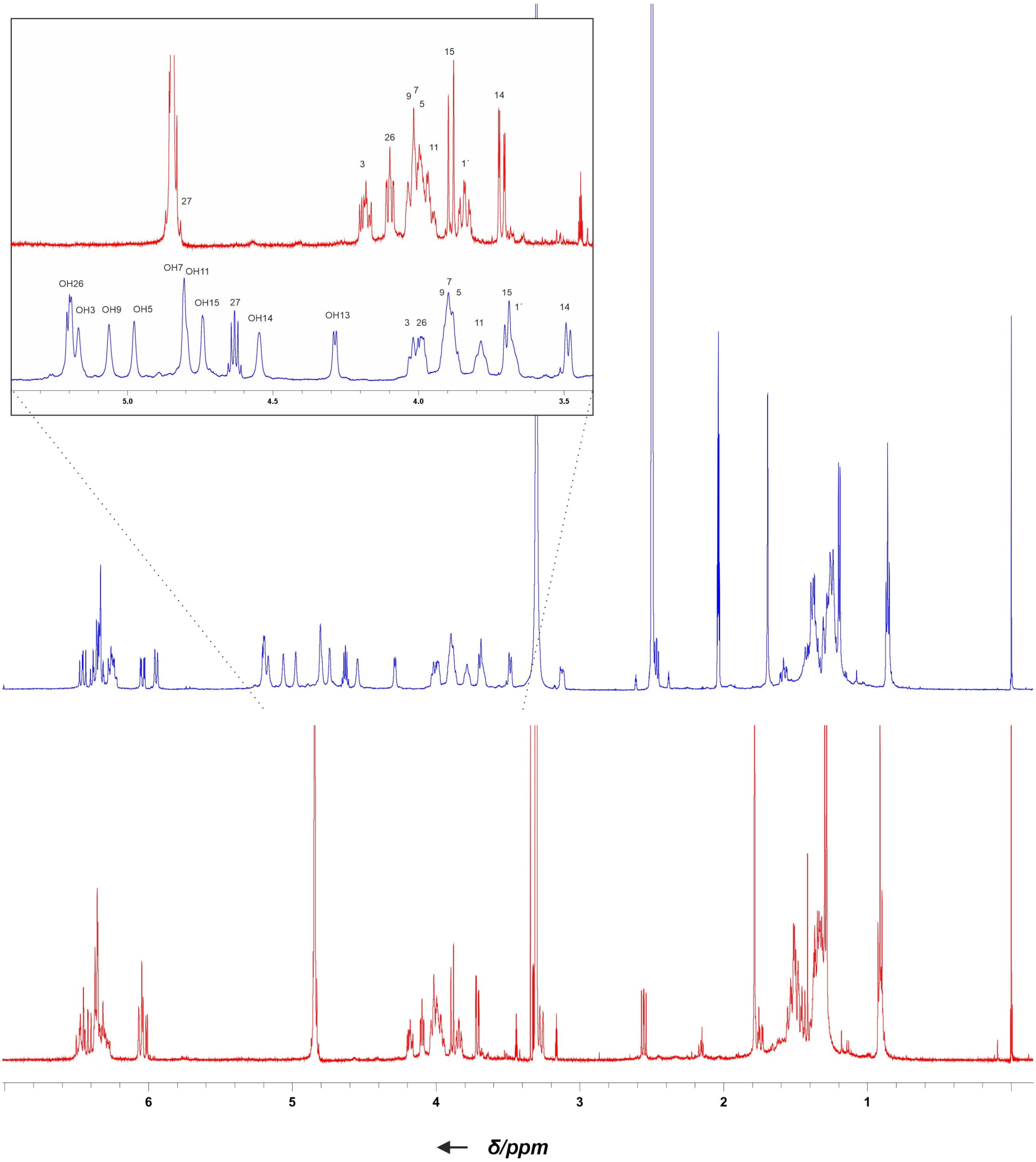

Fig. S5. Comparison of the effect of organic solvent to the quality and line broadening of the NMR spectra. ^{1}H spectrum of pentamycin in deuterated methanol (MeOD) (red) demonstrates that proton signals for several hydroxyl groups are exchanged with the solvent, whereas the signals are visible in DMSO-*d6* (blue).

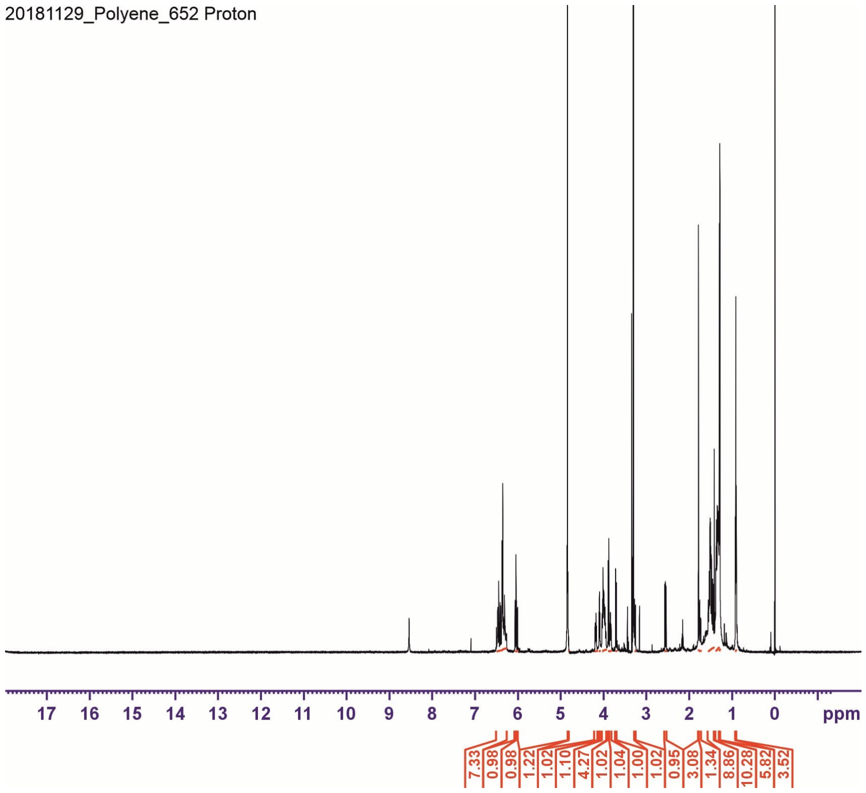

Fig. S6. ^{1}H spectrum of pentamycin in deuterated methanol (MeOD).

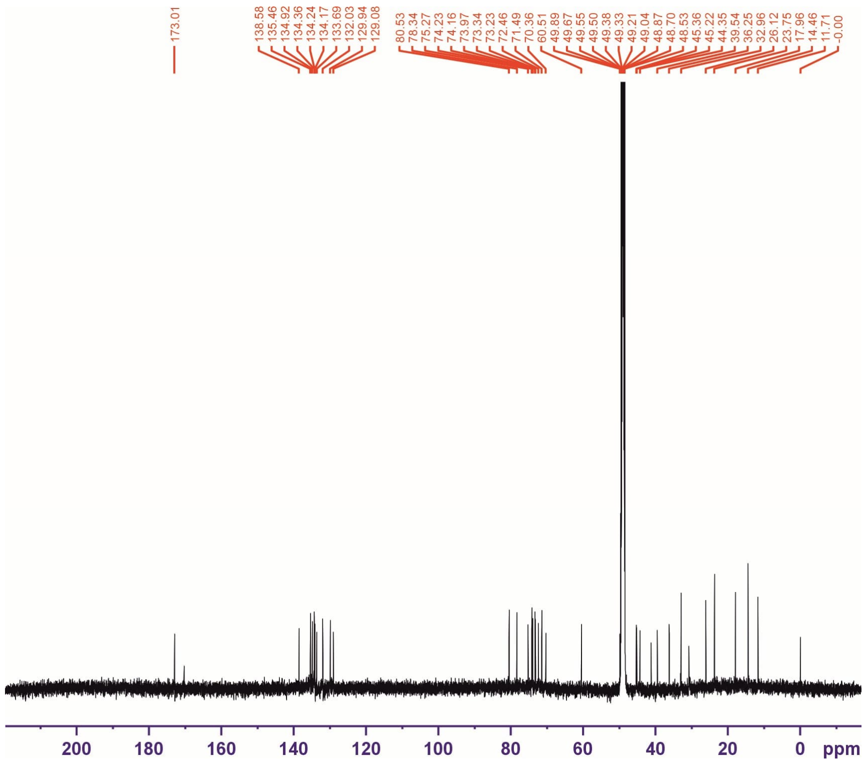

Fig. S7. ^{13}C spectrum of pentamycin in deuterated methanol (MeOD).

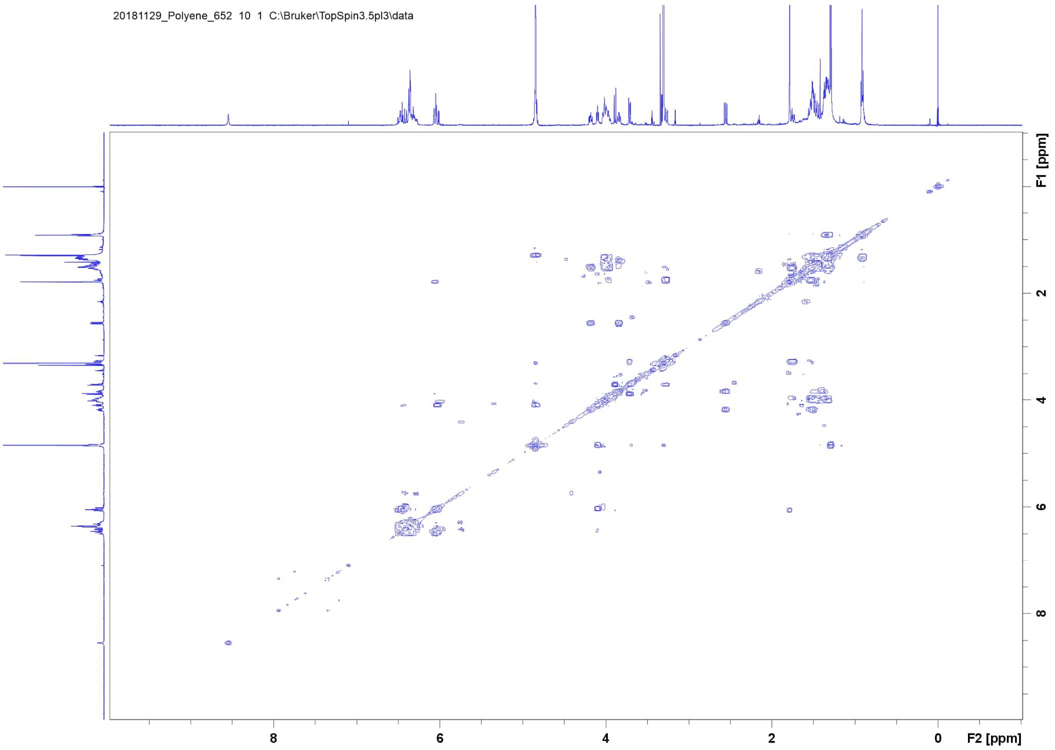

Fig. S8. COSY spectrum of pentamycin in deuterated methanol (MeOD).

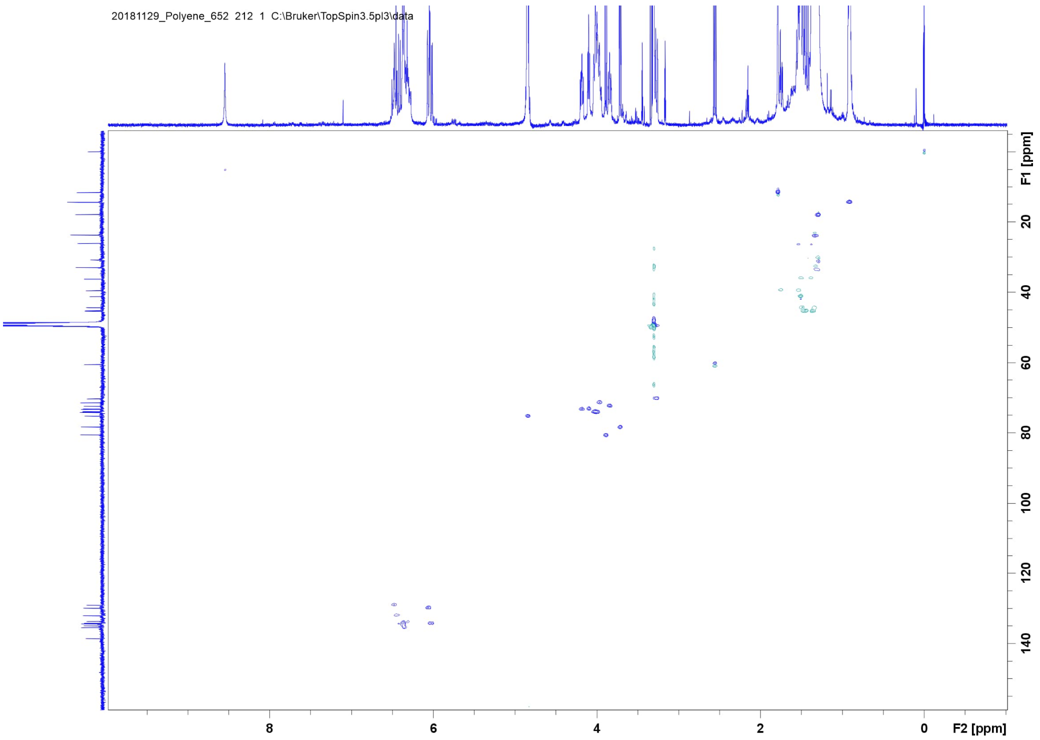

Fig. S9. HSQC spectrum of pentamycin in deuterated methanol (MeOD).

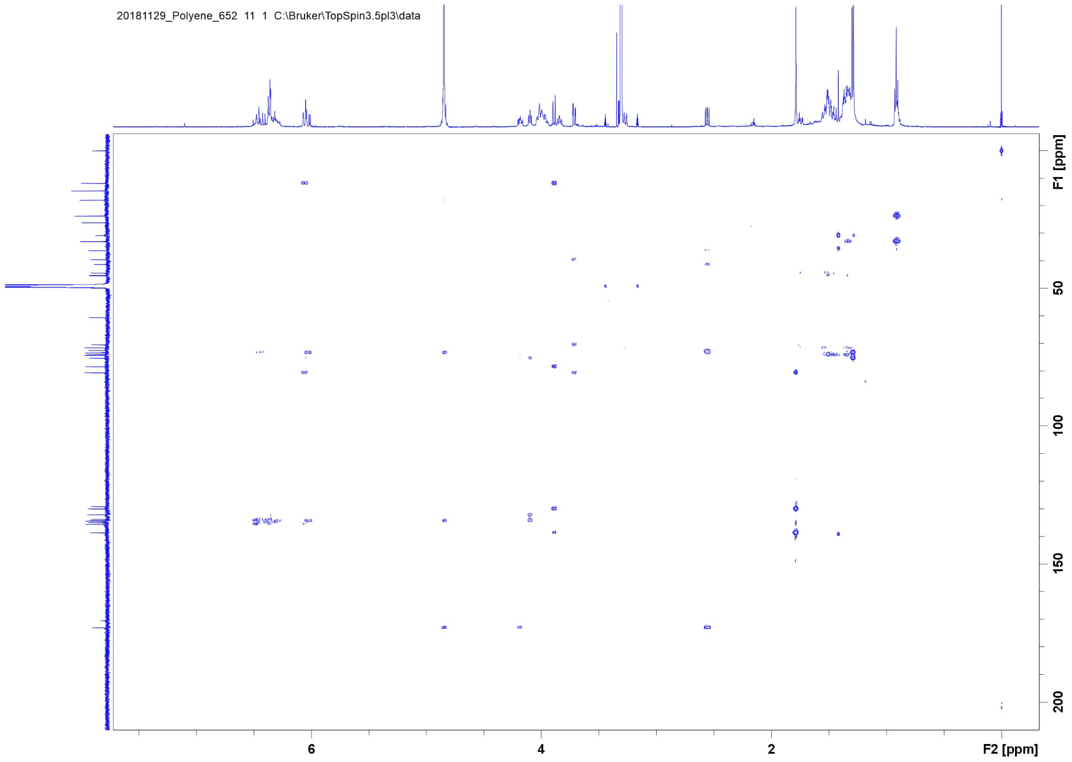

Fig. S10. HMBC spectrum of pentamycin in deuterated methanol (MeOD).

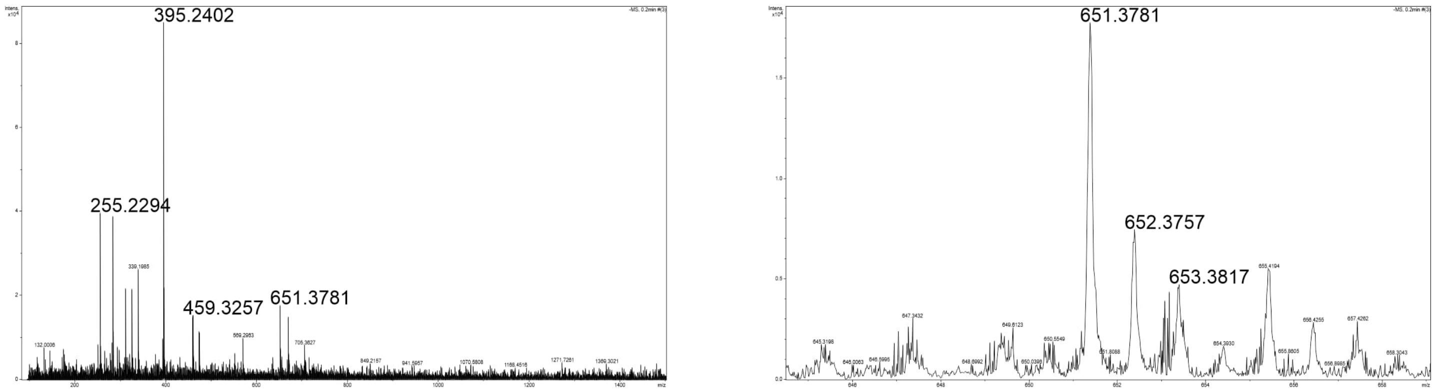

Fig. S11. HR-MS spectrum of pentamycin. $[H\text{-}M\text{-}H]^-$ calc. 651.3783 obs. 651.3781.

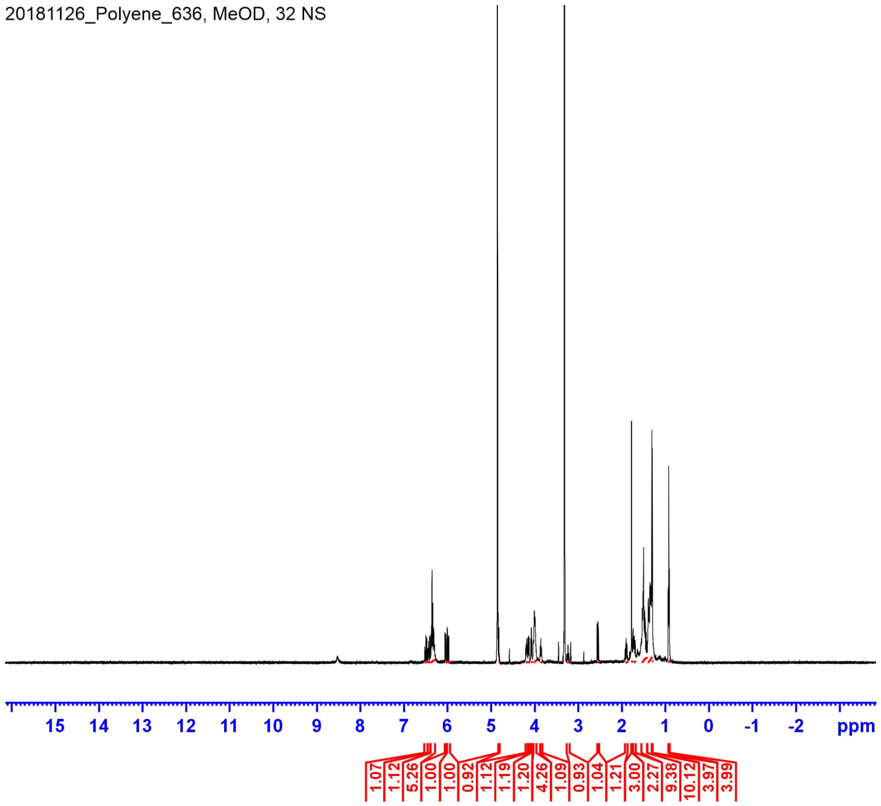

Fig. S12. ^{1}H spectrum of filipin III in deuterated methanol (MeOD).

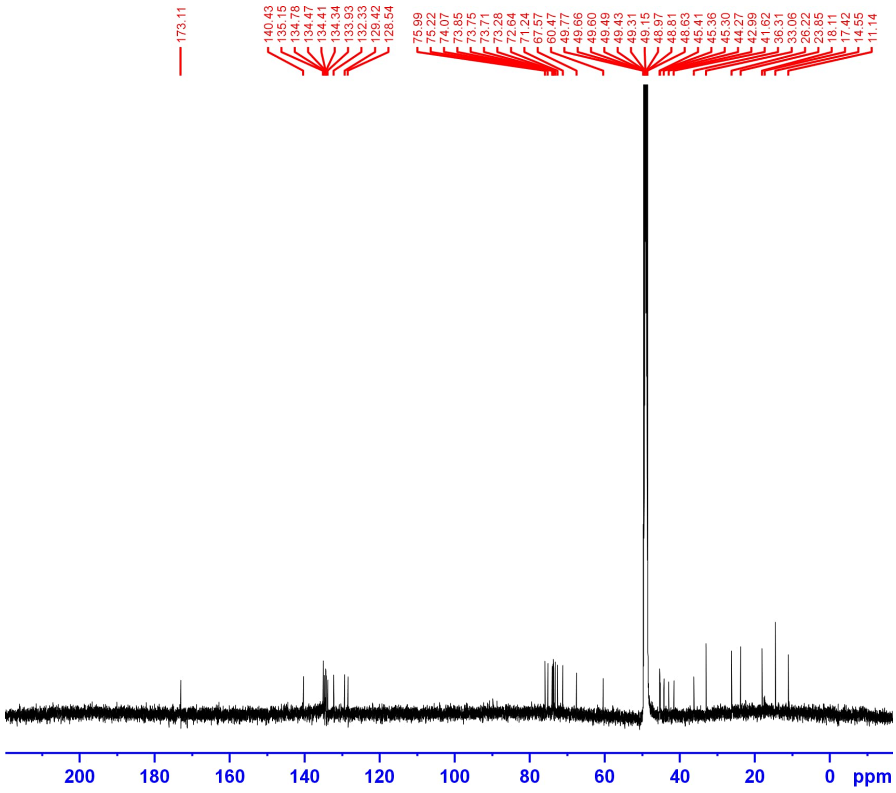

Fig. S13. ^{13}C spectrum of filipin III in deuterated methanol (MeOD).

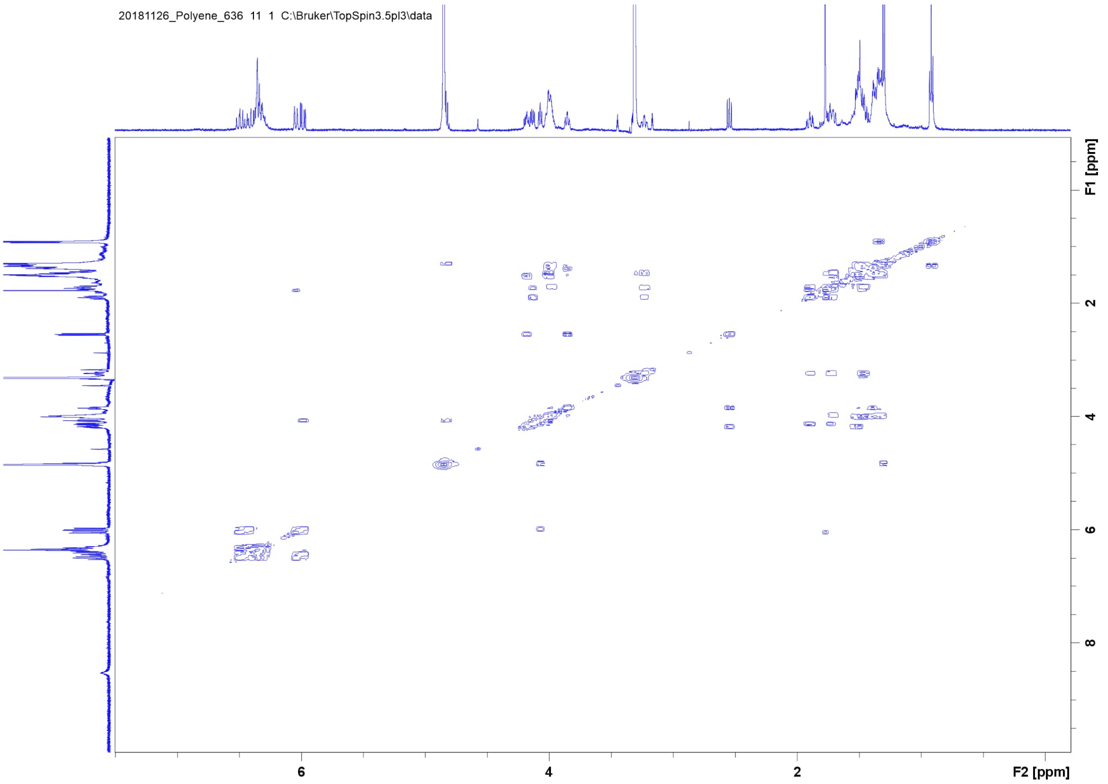

Fig. S14. COSY spectrum of filipin III in deuterated methanol (MeOD).

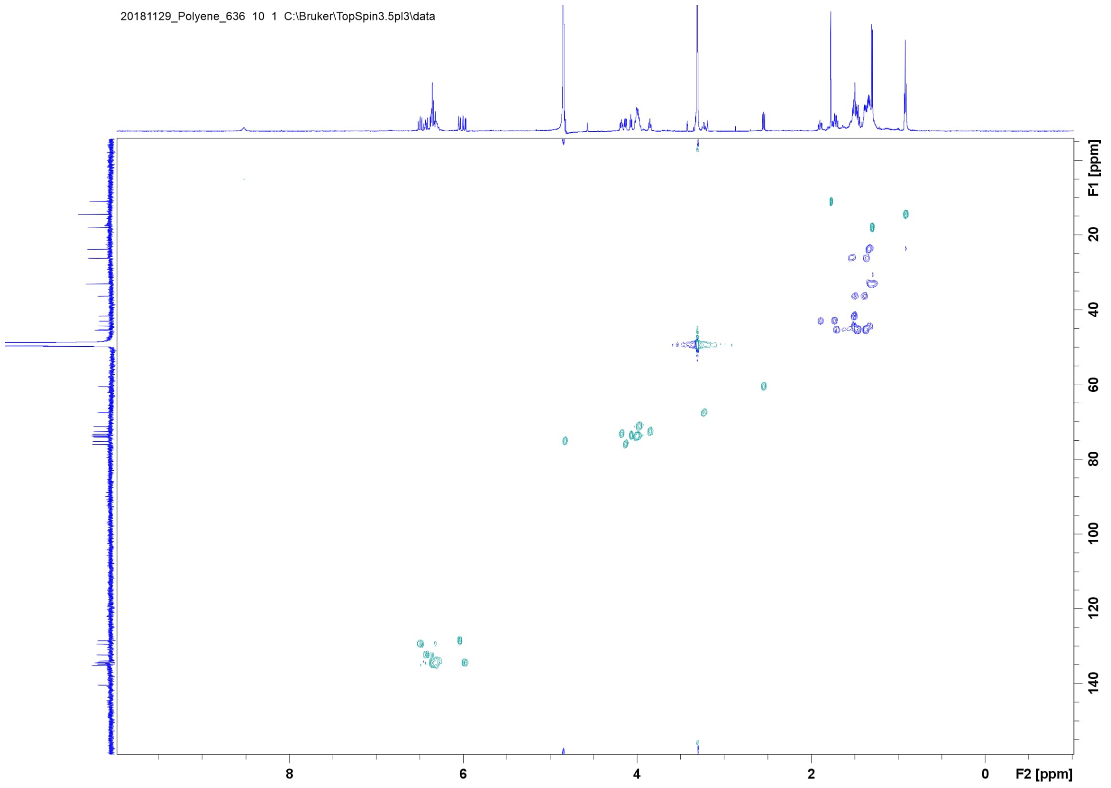

Fig. S15. HSQC spectrum of filipin III in deuterated methanol (MeOD).

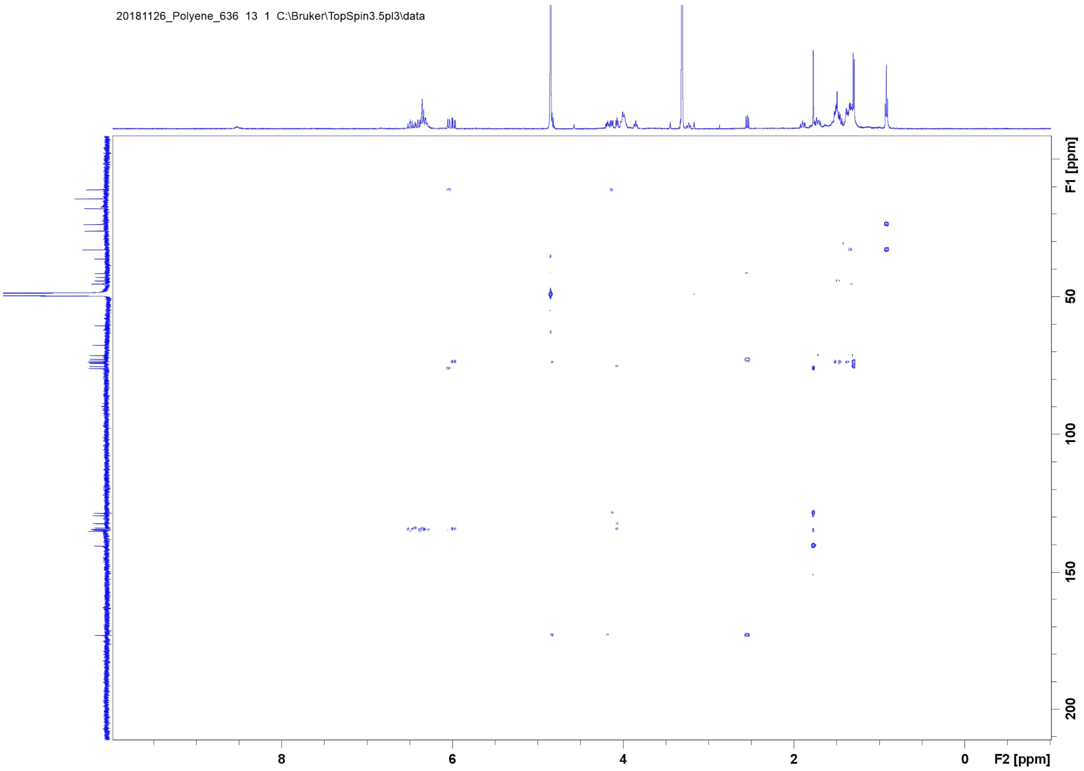

Fig. S16. HMBC spectrum of filipin III in deuterated methanol (MeOD).

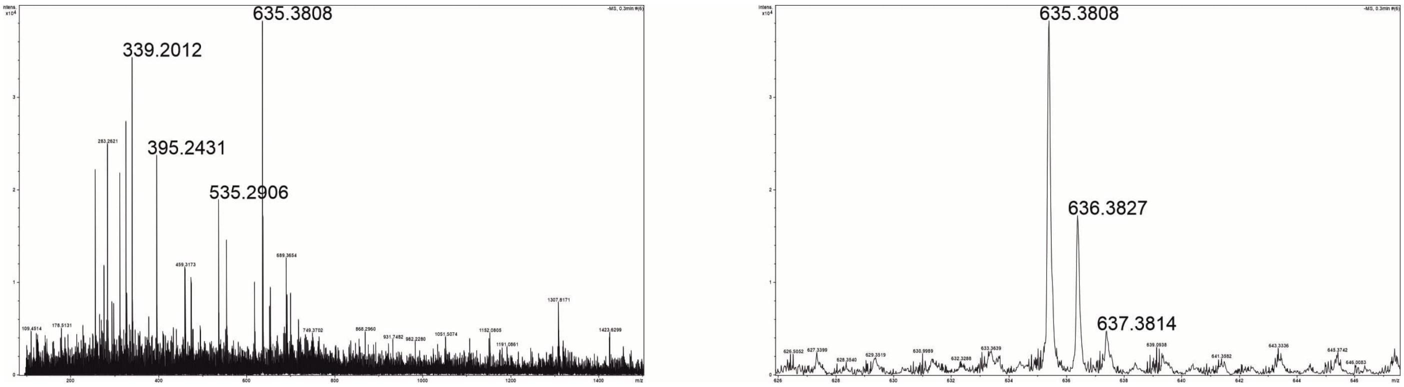

Fig. S17. HR-MS spectrum of filipin III. $[H-M-H]^-$ calc. 635.2958, obs. 635.3808.

Table S1. NMR data for pentamycin in MeOD. * Overlapping with solvent peak, ** Partly overlapping H12 and H29

	δ ppm	δ ppm, *J* Hz
Position	^{13}C	^{1}H
1	173.0	
2	60.5	2.56 dd 7.2, 9.2
3	73.3	4.18 m
4	41.3	1.51 m
5	74.2 (16)	4.01 m
6	45.2	1.48 m
		1.38 m
7	74.0	4.01 m
8	45.4	1.48 m
		1.38 m
9	74.2 (23)	4.01 m
10	44.4	1.5 m
11	71.5	3.97 m
12	39.5	1.54 m
		1.76 ddd 3.5, 10.8, 14.0**
13	70.4	3.27 ddd 1.5, 1.9, 10.8
14	78.3	3.71 dd 1.9, 9.0
15	80.5	3.89 d 9.0
16	138.6	
17	129.9	6.06 dd 1.1, 11.0
18	129.1	6.47 m
19	135.5	6.35 m
20	134.2 (17)	6.36 m
21	134.9	6.36 m
22	133.7	6.36 m
23	134.2 (24)	6.31 m
24	132.0	6.45 m
25	134.4	6.03 dd 4.8, 14.1
26	73.2	4.10 ddd 1.1, 5.5, 6.9
27	75.3	4.84 m*
28	18.0	1.29 d 6.4 3H
29	11.7	1.79 d 1.0 3H**
1'	72.5	3.85 ddd 2.1, 8.9, 9.1
2'	36.3	1.51 m
		1.38 m
3'	26.1	1.53 m
		1.38 m
4'	33.0	1.32 m
5'	23.8	1.33 m
6'	14.5	0.9 t 7.0 3H

Table S2. NMR data for filipin III in MeOD. * Overlapping with solvent peak, ** Partly overlapping H12 and H29

Position	δ ppm ^{13}C	δ ppm, *J* Hz ^{1}H
1	173.1	
2	60.5	2.55 dd 7.4, 9.2
3	73.3	4.18 m
4	41.6	1.51 m
5	73.8	3.99 m
6	45.3	1.47 m
		1.37 m
7	73.7	3.99 m
8	45.4 (37)	1.47 m
		1.37 m
9	74.1	3.99 m
10	44.3	1.52 m
		1.33 m
11	71.2	3.98 m
12	45.4 (41)	1.73 m**
13	67.6	3.23 dddd 1.5, 2.6,
14	43.0	1.90 ddd 3.1, 10.7,
		1.73 m**
15	76.0	4.13 dd 10.7, 4.5
16	140.4	
17	128.5	6.04 dd 11.1, 1.2
18	129.4	6.49 dd 11.3, 14.1
19	135.1	6.35 m
20	134.3	6.35 m
21	134.8	6.35 m
22	133.9	6.35 m
23	134.4	6.35 m
24	132.3	6.43 m
25	134.5	5.98 dd 5.3, 15.0
26	73.75	4.07 ddd 1.1, 5.6, 7.4
27	75.2	4.83 m*
28	18.1	1.30 d 6.3 3H
29	11.1	1.77 d 0.8 3H
1'	72.6	3.85 ddd 2.1, 8.6, 9.3
2'	36.3	1.49 m
		1.39 m
3'	26.2	1.52 m
		1.37 m
4'	33.1	1.33 m
		1.29 m
5'	23.8	1.33 m
6'	14.6	0.92 t 7.0 3H

Table S3. Strains and plasmids used in this study

Strains	Genotype/comments	Source/reference
S. lavendulae YAKB-15	Wild-type	(Yamada *et al*, 2019)
S. galilaeus ATCC 31615	Wild-type	(Kieser *et al*, 2000)
S. albus J1074	Wild-type	(Kieser *et al*, 2000)
S. showdoensis ATCC 15227	Wild-type	(Palmu *et al*, 2017)
S. lividans TK24	Wild-type	(Kieser *et al*, 2000)
S. kanamyceticus DSM 40500	Wild-type	(Zhang *et al*, 2019)
S. candidus NRRL 3601	Wild-type	(Zhao *et al*, 2020)
S. platensis NRRL 8035	Wild-type	(Osada *et al*, 1992)
Saccharomyces cerevisiae BY25610	BY4741ho::Nat-TEFIIpr-mCherry-ADH1ter	Dr. Anssi Malinen
E. coli TOP10	F-mcrA Δ(mrr-hsdRMS-mcrBC) Φ80lacZΔM15 ΔlacX74 recA1 araD139 Δ(araleu)7697 galU galK rpsL (StrR) endA1 nupG	Invitrogen
E. coli ET12567/pUZ8002	*dam-13*::Tn9 *dcm-6 hsdM* pUZ8002	(Kieser *et al*, 2000)
<u>Plasmids</u>		
pBADΔHis	N terminal tail replaced with AHHHHHHHR	(Kallio *et al*, 2006)

Movie S1.

Time-lapse fluorescence microscopy of *S. lavenduale* preying on the *Sacc. cerevisiae.*

Top left: green fluorescence channel; top right: ORCA; bottom left: red fluorescence channel; bottom right: merged channels. Two populations of yeast cells are highlighted in white and purple boxes. The population in the white box remained unaffected by *S. lavendulae*, while the population in the purple box interacted with *S. lavendulae*.

Movie S2.

Close-up of the bottom right panel (merged channels) from Movie S1.

Data S1. (separate file)

Extracellular proteome analysis of *S. lavendulae* axenic culture and in co-culture with *Sacc. cerevisiae*.

References

Kallio P, Sultana A, Niemi J, Mäntsälä P & Schneider G (2006) Crystal Structure of the Polyketide Cyclase AknH with Bound Substrate and Product Analogue: Implications for Catalytic Mechanism and Product Stereoselectivity. *Journal of Molecular Biology* 357: 210–220

Kieser T, Bibb MJ, Buttner MJ, Chater KF & Hopwood DA (2000) Practical streptomyces genetics John Innes Foundation Norwich

Osada H, Koshino H, Kudo T, Onose R & Isono K (1992) A new inhibitor of protein kinase C, RK-1409 (7-oxostaurosporine). I. Taxonomy and biological activity. *J Antibiot* 45: 189–194

Palmu K, Rosenqvist P, Thapa K, Ilina Y, Siitonen V, Baral B, Mäkinen J, Belogurov G, Virta P, Niemi J, *et al* (2017) Discovery of the Showdomycin Gene Cluster from *Streptomyces showdoensis* ATCC 15227 Yields Insight into the Biosynthetic Logic of C-Nucleoside Antibiotics. *ACS Chem Biol* 12: 1472–1477

Yamada K, Koroleva A, Laughlin M, Oksanen N, Akhgari A, Safronova V, Yakovleva E, Kolodyaznaya V, Buldakova T & Metsä-Ketelä M (2019) Characterization and overproduction of cell-associated cholesterol oxidase ChoD from Streptomyces lavendulae YAKB-15. *Scientific Reports* 9: 11850

Zhang S, Chen T, Jia J, Guo L, Zhang H, Li C & Qiao R (2019) Establishment of a highly efficient conjugation protocol for *Streptomyces kanamyceticus* ATCC12853. *MicrobiologyOpen* 8

Zhao G, Yao S, Rothchild KW, Liu T, Liu Y, Lian J, He H, Ryan KS & Du Y (2020) The Biosynthetic Gene Cluster of Pyrazomycin—A C-Nucleoside Antibiotic with a Rare Pyrazole Moiety. *ChemBioChem* 21: 644–649
